# Structural and dynamic studies of DNA recognition by NF-κB p50 RHR homodimer using methyl NMR spectroscopy

**DOI:** 10.1093/nar/gkac535

**Published:** 2022-06-24

**Authors:** Amrinder Singh, Maria A Martinez-Yamout, Peter E Wright, H Jane Dyson

**Affiliations:** Department of Integrative Structural and Computational Biology, Scripps Research, 10550 North Torrey Pines Road, La Jolla CA 92037, USA; Department of Integrative Structural and Computational Biology, Scripps Research, 10550 North Torrey Pines Road, La Jolla CA 92037, USA; Department of Integrative Structural and Computational Biology, Scripps Research, 10550 North Torrey Pines Road, La Jolla CA 92037, USA; Department of Integrative Structural and Computational Biology, Scripps Research, 10550 North Torrey Pines Road, La Jolla CA 92037, USA

## Abstract

Protein dynamics involving higher-energy sparsely populated conformational substates are frequently critical for protein function. This study describes the dynamics of the homodimer (p50)_2_ of the p50 Rel homology region (RHR) of the transcription factor NF-κB, using ^13^C relaxation dispersion experiments with specifically (^13^C, ^1^H)-labeled methyl groups of Ile (δ), Leu and Val. Free (p50)_2_ is highly dynamic in solution, showing μs-ms relaxation dispersion consistent with exchange between the ground state and higher energy substates. These fluctuations propagate from the DNA-binding loops through the core of the domain. The motions are damped in the presence of κB DNA, but the NMR spectra of the DNA complexes reveal multiple local conformations of the p50 RHR homodimer bound to certain κB DNA sequences. Varying the length and sequence of κB DNA revealed two factors that promote a single bound conformation for the complex: the length of the κB site in the duplex and a symmetrical sequence of guanine nucleotides at both ends of the recognition motif. The dynamic nature of the DNA-binding loops, together with the multiple bound conformations of p50 RHR with certain κB sites, is consistent with variations in the transcriptional activity of the p50 homodimer with different κB sequences.

## Graphical Abstract

**Graphical Abstract:**
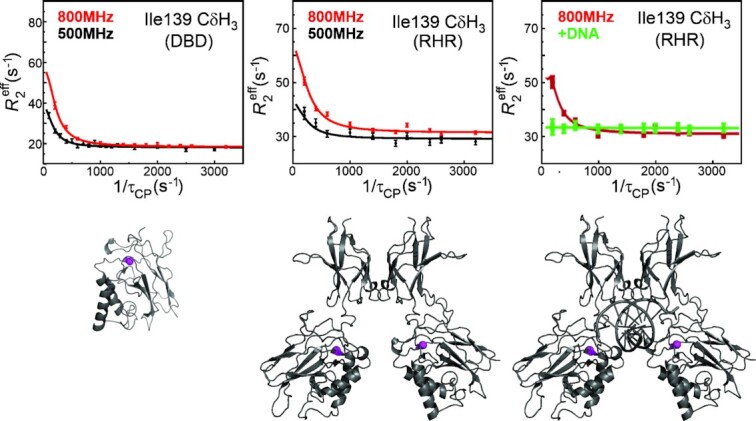
Relaxation dispersion profiles for Ile 139 CδH3 in p50 DBD and p50 RHR dimer, and after addition of DNA (right). Ile 139 in each structure is shown in the lower panels.

## INTRODUCTION

The nuclear factor-κB (NF-κB) family of transcription factors initiates the expression of a wide variety of genes that are involved in many biological processes, varying from immune, stress, and inflammatory responses, to cell apoptosis (1,2). The entire family of mammalian NF-κB transcription factors is encoded by five genes: *NFKB1*, *NFKB2*, *REL A*, *REL B* and *REL* (3). Proteolysis of their products provides functional DNA-binding polypeptide subunits p50, p52, RelA (p65), RelB and c-Rel respectively, all of which share a ∼300 amino acid DNA binding and dimerization domain, known as the Rel homology region (RHR). The five subunit proteins combine to produce 15 NF-κB homo- and heterodimers with distinct functions. The best-known NF-κB dimer is the p50–p65 heterodimer, which is sequestered in the cytoplasm in resting cells in complex with the inhibitory protein IκBα (4). The latent NF-κB:IκBα cytoplasmic complex can be activated by various extracellular stimuli, through the action of IκB kinase (IKK), which phosphorylates IκBα. Subsequent ubiquitination and degradation of IκBα releases free NF-κB heterodimer, which then rapidly accumulates in the nucleus and binds to κB DNA sites, elevating their expression (5).

**Figure 1.**
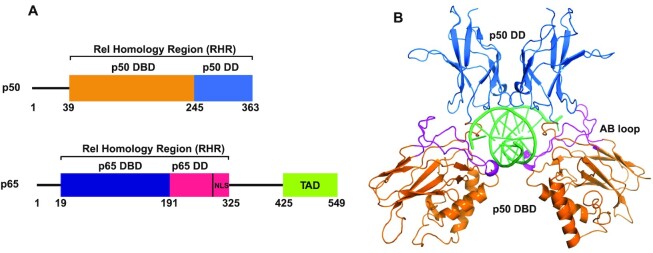
(**A**) Schematic representation of domain organization of the p50 and p65 subunits of NF-κB; DBD: DNA binding domain; DD: dimerization domain; NLS: nuclear localization sequence; TAD: C-terminal trans-activation domain. (**B**) Cartoon representation of X-ray crystal structure of p50 RHR in complex with a 10 base-pair DNA duplex (PDB: 1NFK) (25). DNA in green, DBD in orange, DD in blue. The AB loop (residues 49–80) is shown in magenta.

The p50–p65 heterodimer promotes expression of target genes through the presence of the C-terminal transactivation domain of p65. In contrast, the p50 RHR homodimer (p50)_2_ (Figure 1) lacks a transactivation domain and acts as a repressor of transcriptional activity (6). In unstimulated cells, high basal levels of p50 homodimer may compete for DNA binding with transcriptionally-active NF-κB dimers, particularly the p65/p50 heterodimer (7). However, (p50)_2_ has also been found to be transcriptionally active with some κB motifs, but not with others (8). Understanding the dynamic and structural basis of the regulation of NF-κB–DNA interactions provides important insights into the various functions undertaken by different members of the NF-κB family.

Nuclear magnetic resonance (NMR) spectroscopy provides an unparalleled opportunity to investigate both structural changes and polypeptide chain dynamics. To elucidate dynamics on the physiologically-relevant μs-ms time scale, we use Carr-Purcell-Meiboom-Gill (CPMG) relaxation dispersion experiments to provide information about both the kinetics and the thermodynamics of the exchange process, together with an estimation of the structural changes involved (9,10). Briefly, in CPMG relaxation dispersion experiments, the effective transverse relaxation rate (*R*_2,eff_) is measured as a function of refocusing pulse frequency (ν_CPMG_). As the intervals between the refocusing pulses are varied, the effects of conformational exchange are quenched, providing dispersion profiles. Typical relaxation dispersion profiles, when fit to an appropriate model of exchange, provide information on the chemical exchange process, in the form of estimates of the excited-state population (*p*_B_), interconversion rate (*k*_ex_), and structural features of the excited state, in the form of chemical shift differences between the ground and excited states.

Traditionally, measurement of polypeptide dynamics by NMR involves studying the motions of the protein backbone, using ^15^N relaxation measurements and ^15^N-based relaxation dispersion experiments (11). However, for larger dynamic complexes, amide resonances are frequently broadened beyond detection, even when transverse relaxation is optimized in TROSY spectra, due to exchange processes. Recent experimental developments have made it possible to probe both ps-ns and μs-ms timescale motions using specifically labeled isolated ^13^CH_3_ groups in large complexes (12). Methyl groups have favorable relaxation properties compared to amides; they are excellent reporters of dynamics in proteins and are well-suited for applications involving high molecular weight systems. Nowadays, robust approaches are available to produce highly deuterated proteins specifically labeled at the methyl groups of Ile (*δ*1), Leu and Val with ^13^C and ^1^H (13).

In the current work, methyl CPMG relaxation dispersion experiments performed at multiple field strengths show that the free form of the NF-κB p50 RHR homodimer exists in a dynamic equilibrium between the ground state and one or more sparsely populated higher energy excited states; the interconversion occurs on a millisecond timescale. Addition of a DNA duplex containing a κB sequence causes damping of these motions. To elucidate the influence of local variations in DNA sequence, we used methyl HMQC NMR spectroscopy to investigate the binding of the p50 RHR homodimer with various κB DNA sites and identified a set of κB sequences that perturb the methyl NMR spectrum of the p50 RHR. Addition of several of these duplexes gave spectra that indicated the presence of multiple conformations of the complex. We conclude that both the length and symmetry of the duplex are important factors in the interaction of κB DNA sequences with the p50 RHR homodimer, and that the presence of a single local bound conformation, rather than multiple conformations, is correlated with κB sequences that are capable of transcriptional activation mediated by the p50 homodimer.

## MATERIALS AND METHODS

### Reagents

#### Protein expression and purification

Plasmids containing various lengths of the RHR of murine p50 were prepared by subcloning from the full-length genes. The p50 DNA-binding domain (DBD) and dimerization domain (DD) contained residues 39–247 and 245–350 respectively; the p50 RHR contained residues 39–363. Expression of ^15^N labeled p50 DBD and p50 DD was carried out as described previously (14). U-[^2^H, ^15^N] labeled p50 RHR homodimer (39–363) was over-expressed in *Escherichia coli* BL-21(DE3) cells in 1 l of D_2_O M9 minimal medium supplemented with 4 g/l of ^12^C glucose and 1 g/l ^15^N-labeled ammonium chloride as sole source of carbon and nitrogen respectively. The culture was induced with 0.1 mM final concentration of IPTG at optical density (600 nm) of 0.2 at 37°C followed by overnight incubation with agitation at 21°C.

Selectively methyl protonated [Ile (*δ*1 only), Leu (^13^CH_3_, ^12^CD_3_), Val (^13^CH_3_,^12^CD_3_)] U-[^15^N, ^12^C, ^2^H] samples (termed ILV) (15) of p50 DBD, p50 DD and p50 RHR were obtained by protein overexpression from a culture of *Escherichia coli* BL21(DE3) cells transformed with the respective plasmids. The proteins were expressed in 1 l of D_2_O M9 medium using 3 g/l of U-[^12^C,^2^H]-glucose as the main carbon source and 1 g/l of ^15^NH_4_Cl as the nitrogen source. One hour prior to induction, 120 mg of 2-keto-3,3-*d*2-1,2,3,4–^13^C-butyrate and 200 mg of 2-keto-3-methyl-*d*3-3-*d*1-1,2,3,4–^13^C-butyrate were added to the growth medium. Incubation was continued for 22 h at 21°C. The details of the subsequent purification steps for U-^15^N labeled and ILV labeled p50 DBD and p50 DD were described previously (14). For [^2^H, ^15^N] labeled and ILV labeled p50 RHR homodimer the cells were harvested after 20 h growth at 20°C by centrifuging at about 4000 rpm for 20 min, and suspended in a lysis buffer containing 20 mM Tris (pH 7.5), 50 mM NaCl, 10% glycerol, 2 mM DTT, 0.5 mM EDTA and a protease inhibitor cocktail tablet (Pierce EDTA free). Cells were lysed by sonication. Following centrifugation, the supernatant containing the soluble fraction of the protein was collected and loaded onto an anion-exchange chromatography column containing Q-Sepharose Fast Flow beads (GE Healthcare) and followed by 50 ml wash buffer (lysis buffer minus protease inhibitor cocktail tablet). The flow-through from the Q column was loaded onto a SP-Sepharose cation-exchange resin column and washed with 100 ml wash buffer. The labeled p50 RHR homodimer was eluted with elution buffer (20 mM Tris pH 7.5, 200 mM NaCl, 0.5 mM EDTA and 2 mM DTT). The eluted fractions were checked on SDS-PAGE gel and the fractions containing protein were pooled and dialyzed with 20 mM Tris (pH 7.5), 50 mM NaCl, 0.5 mM EDTA and 2 mM DTT. The dialyzed protein was filtered and loaded onto a 5 ml HiTrap SP-column pre-equilibrated with 20 mM Tris pH 7.5, 50 mM NaCl and 2 mM DTT. The protein was eluted on an Akta purifier with a salt gradient of 50 mM NaCl to 300 mM NaCl in 100 min at a rate of 3 ml/min. Identity and purity of the protein was confirmed by SDS-PAGE. The fractions containing the p50 RHR homodimer were pooled and concentrated and subjected to size exclusion chromatography using a Superdex-75 column. The pure fractions were concentrated, and buffer exchanged into NMR buffer (20 mM Tris pH 6.8, 150 mM NaCl, 2 mM DTT) using a NAP-5 column.

#### DNA oligonucleotides

DNA sequences containing varying κB sites were purchased from IDT. Duplex DNA for NMR experiments was prepared through self-annealing of the single stranded DNA by heating at 95°C followed by gradual cooling to room temperature in annealing buffer (50 mM Tris pH 8.0, 150 mM NaCl). DNA concentration was determined by UV absorbance at 260 nm. For DNA sequences containing two different strands, the concentration of each strand was measured by UV absorbance at 260 nm and the strands were then mixed in a 1:1 ratio for annealing using the same conditions as mentioned above.

#### Characterization of p50 RHR homodimer and its DNA complexes

The preparation and characterization of the p50 homodimer has been described previously (14). The complexes of the p50 homodimer with oligonucleotides containing various κB sequences were prepared by addition of an equimolar amount of DNA duplex to the solution of p50 homodimer in NMR buffer. The complex of p50 RHR with each of the DNA duplexes was shown to be a single homogeneous species using native gel electrophoresis (16).

### Biological resources

#### *Escherichia coli* BL21(DE3)

Plasmid pET21b containing the gene for p50 DBD (residues 39–247) or p50 DD (residues 245–350) or the p50 RHR (residues 39–363).

### Spectroscopic methods

#### NMR spectroscopy

**Figure 2.**
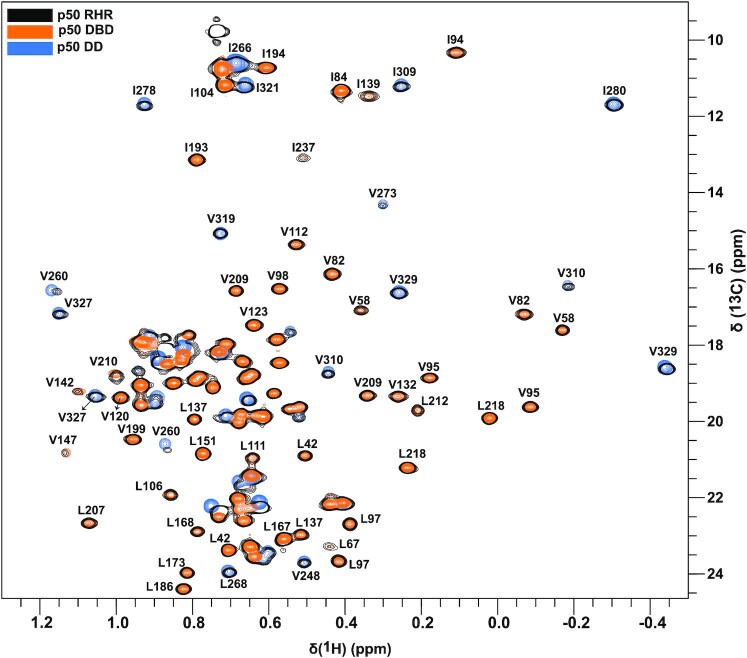
Overlay of ^13^C methyl HMQC spectra of p50 RHR (black) drawn with 5 contour levels, p50 DBD (orange) and p50 DD (blue). Selected assignments (14) are labeled. Fully labeled ^13^C methyl HMQC spectra of p50 DBD and DD are shown in Supplementary Figure S3.

NMR spectra (^1^H–^15^N HSQC for p50 DBD and DD and TROSY for the p50 RHR homodimer) were acquired on a Bruker Avance III NMR spectrometer operating at a magnetic field strength of 18.8 T, corresponding to an 800 MHz proton Larmor frequency, equipped with a Bruker TXI cryo-probe or on a Bruker Avance spectrometer operating at a magnetic field strength of 21.1 T, corresponding to a 900 MHz proton Larmor frequency. The measurements were carried out at 300 K in NMR buffer (20 mM Tris pH 6.8,150 mM NaCl, 2 mM DTT). Resonance assignments for the RHR and the DNA complexes were inferred from those of the individual domains, as previously described (17). The size of the RHR and the DNA complexes precludes acquisition of triple resonance data that would be needed for the direct assignment of resonances in these systems. Assignments for these systems are inferred from the coincidence of cross peaks in the shorter and longer constructs (for RHR) (see Figure 2) and between the free RHR and the DNA complexes (see Figure 6). Where the cross peaks of the DNA complex are not coincident with those of the free protein (and also in the case of duplicates) we have inferred the identity of the shifted cross peaks from their proximity to the peak in the spectrum of the free protein. This process gains validity from the small number of shifted cross peaks (most of the surrounding cross peaks are coincident between free and bound spectra) and from the consistency of the results between complexes with different DNAs. The assignment of the L67 cross peaks in Area 2 of Figure 6 may give rise to some uncertainty – we assigned the shifted cross peak of V248 to the same position in the three panels, and subsequently assigned the shifted and duplicated cross peaks of L67 and L97 to the remaining cross peaks.

#### Methyl CPMG relaxation dispersion experiments

**Figure 3.**
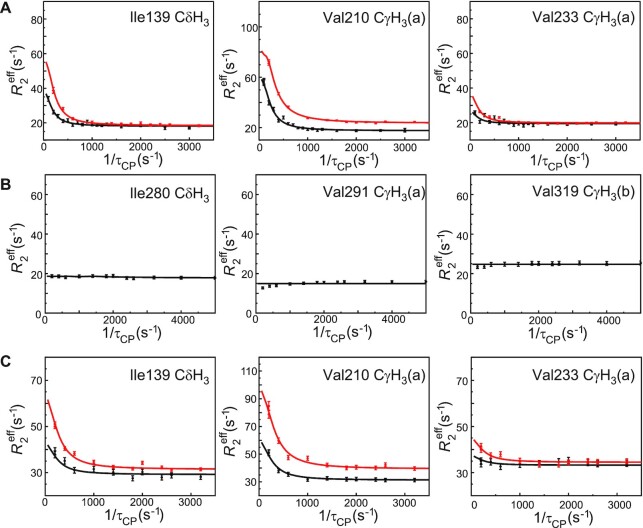
Representative methyl R_2_ relaxation dispersion profiles for p50. (**A**) p50 DBD at 300 K, pH 6.8. Black and red, methyl ^13^C dispersion data at 11.7 T and 18.8 T, respectively. (**B**) p50 DD at 300 K, pH 6.8, 18.8 T spectrometer. (**C**) p50 RHR homodimer 11.7 T, black; 18.8 T, red. Solid lines represent fits to the individual data sets using the program Glove (20). The designation (a) or (b) refers to the two prochiral methyl groups of Leu and Val, which have not been stereospecifically assigned. Complete sets of all resolvable relaxation data profiles are shown in Supplementary Figures S4 and S5.

Methyl single-quantum ^13^C CPMG relaxation dispersion experiments (12) were recorded on highly deuterated ILV-methyl labeled p50 DBD, p50 DD and p50 RHR samples at field strengths of 11.7 T and 18.8 T (500 and 800 MHz), at 300K, using Bruker spectrometers. The CPMG data were acquired as pseudo 3D experiments with a constant relaxation time period *T*_relax_ of 20 ms and with 18 CPMG refocusing pulse frequencies ν_CPMG_ = 1/(2*t*), where *t* is the delay between the consecutive 180^o^ refocusing pulses in ^13^C CPMG pulse-train. An interscan delay of 1.5 s was used with 24, 32 or 36 scans/FID, giving rise to net acquisition times between 40–58 h for a complete pseudo-3D data set. All data were processed using NMRPipe (18) and peak intensities were measured using CCPN (19). Data points were plotted as the effective transverse relaxation rate *R*_2,eff_ versus 1/τ_CP_. Error bars on data points in Figures 3, 8, Supplementary Figure S4, S5, S7 and S11 were estimated using duplicate points collected for the reference CPMG experiment and for the data points at 1/τ_CP_ = 200 and 6000 s^−1^. Plots are shown only up to 3000 s^−1^ as the data points were flat between 3000 and 6000 s^−1^. The variation in *R*_2,eff_ with ν_CPMG_ was fitted to the Bloch–McConnell equations for the two-site exchange model using the program Glove (20) to extract values of exchange parameters, as well as ^13^C chemical shift differences for nuclei interconverting between pairs of states. Uncertainties in the parameters were estimated using Monte Carlo simulations.

## RESULTS

### NMR characterization of full length p50 RHR homodimer

Backbone and side chain assignments of each of the two domains of p50 RHR (DBD and DD) were recently reported (14,21). In a previous NMR study of full length p65 RHR (17), the assignments of the full-length RHR (in the heterodimer with deuterated, ^14^N, ^12^C p50 RHR) were derived from those of the individual domains, since the DBD and DD domains of p65 do not interact with each other in the free protein. A similar strategy has been applied here to the labeled p50 homodimer to transfer both the backbone (Supplementary Figure S1) and methyl assignments (Figure 2) to the full length p50 RHR homodimer from the individual domains. The ^1^H–^15^N HSQC spectrum of p50 DBD contains a considerable number of missing amide cross peaks (20%) at 300K. These resonances are presumably broadened beyond detection by chemical exchange between different conformational states in the free p50 DBD. The same set of residues, mostly located in the DNA recognition loop region, are also missing from the ^1^H–^15^N transverse relaxation optimized spectroscopy (TROSY) spectrum of full-length p50 RHR homodimer in the free state. The ^15^N line broadening leading to the loss of amide resonances in the DNA recognition loop suggests that the loop is flexible, sampling two or more backbone conformations with different chemical environments on an intermediate (μs-ms) timescale. Consistent with this hypothesis, many of the amide resonances that are missing or very weak in an HSQC spectrum at 300K gain intensity as the temperature is lowered to 290 K (Supplementary Figure S2A). The locations of the residues with missing or weak cross peaks in the ^15^N TROSY spectrum of free p50 RHR are mapped onto the structure of the DNA complex in Supplementary Figure S2B. It should be noted that a high-resolution crystal structure of the free protein is not available, likely because its flexibility precludes the formation of crystals.

### Conformational exchange revealed by methyl CPMG relaxation dispersion experiments

The p50 RHR homodimer is a 74 kDa multi-domain protein whose size challenges traditional solution-state NMR approaches for studying its structure and dynamics. Based on our observation that the ^1^H–^15^N TROSY spectrum of the p50 RHR homodimer is very complex and contains broadened amide resonances (Supplementary Figure S1C), studying its backbone dynamics using conventional ^15^N relaxation dispersion experiments is not feasible. To alleviate the problem of exchange broadening of NH resonances, we exploited the high sensitivity of methyl groups in a highly deuterated environment. Specific methyl labeling of isoleucine, leucine, and valine (ILV labeling) is ideal for probing complex systems, as these methyls are relatively sparse and are often close to sites of interest in the interior of the protein. To gain insights into conformational changes on p50 RHR homodimer in solution using methyl CPMG relaxation dispersion experiments, we began by characterizing dynamics in the isolated ILV-labeled p50 DBD (25 kDa) and DD (12 kDa × 2 = 24 kDa dimer), a similar strategy to that used for the resonance assignments. The domains were prepared as highly deuterated, selectively Ile ^13^Cδ1; Leu ^13^Cδ1/^13^Cδ2; Val ^13^Cγ1/^13^Cγ2-labeled samples, which give rise to well-dispersed 2D methyl HMQC spectra (Supplementary Figure S3). The methyl HMQC spectra of the DBD and DD domains are shown superimposed on that of the p50 RHR homodimer in Figure 2.

Single-quantum ^13^C-methyl relaxation dispersion experiments (12) were performed on ILV-labeled p50 DBD and DD. Figure 3A, B show representative relaxation dispersion data for the DBD and DD domains. A complete set of R_2_ dispersion profiles for these domains is shown in Supplementary Figure S4. Interestingly, we observe evidence for extensive μs-ms time scale motions in the p50 DBD (Figure 3A, Supplementary Figure S4A), but none at all in the DD (Figure 3B, Supplementary Figure S4B). The absence of dynamics in the dimerization domain corroborates earlier observations of backbone ^15^N order parameters close to 1 for the p50 DD, an indication that it is highly rigid on the ps-ns time scale (22).

Next, we investigated the conformational dynamics of full length ILV p50 RHR homodimer to determine whether the dynamics prevailing in an isolated p50 DNA binding domain also occur in the full-length protein. We asked whether the rigidity of the DD would damp out the dynamics in the DBD, or whether the DBD dynamics would influence the DD towards greater local flexibility. Methyl CPMG relaxation dispersion data acquired with ILV-labeled full-length p50 RHR homodimer (Figure 3C and Supplementary Figure S5) showed that the motions present in the isolated DBD were also present for the same residues in the full-length p50 RHR homodimer, and that the p50 DD remains rigid in the full-length protein. These observations are consistent with the lack of contact between the DBD and DD domains of all of the free NF-κB RHRs so far studied (23).

**Figure 4.**
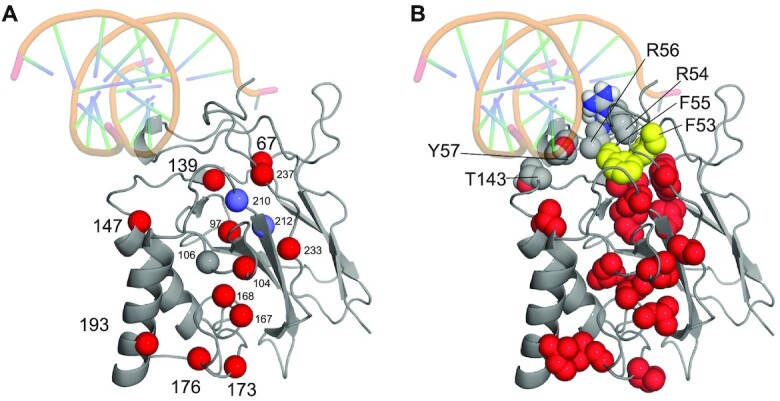
Mapping of the dispersing residues in the DBD free p50 RHR onto a portion of the X-ray crystal structure of the p50 RHR DNA complex (PDB 1NFK) (25). (**A**) Cα positions of residues with dispersing methyl-group resonances (red spheres: cluster 1, blue spheres: cluster 2). (**B**) Side chains of dispersing residues are shown in red spheres, together with those of the DNA-binding residues and the two phenylalanines that connect the DNA-binding loop to the hydrophobic spine containing the dispersing residues. Other DNA contact residues include Y57 and T143 as shown. The DNA was not present in the experiment but is included to show the position of the DNA-binding loops.

Methyl peaks from 18 (p50 DBD) and 19 (p50 RHR) residues showed conformational exchange dynamics with *R*_ex_ (the vertical displacement of the dispersion curve from high 1/τ_CP_ to low 1/τ_CP_) > 5 s^−1^ (Supplementary Figures S4A, S5). Most of the dispersion curves can be fitted by a simple two-site exchange process. The dispersion profiles for individual residues were initially fitted independently. The data were then clustered on the basis of the *k*_ex_ values derived from the individual fits. Residues were grouped into clusters using the criterion χ^2^_cluster_/χ^2^_individual_ < 2, where χ^2^_individual_ is the reduced χ^2^ value for a given residue when its dispersion profile is fitted independently and χ^2^_cluster_ is the reduced χ^2^ value for that residue when fitted to a common *k*_ex_ and p_B_ shared by all residues in the cluster. For the isolated DBD, all of the residues showing dispersion, except Leu106, Ile139, Val210, and Leu212, were consistent with a single cluster with *k*_ex_ = 1640 ± 47 s^−1^ and p_B_ = 4.2 ± 0.1%. Ile139, Val210, and Leu212 fitted a second cluster with *k*_ex_ = 420 ± 37 s^−1^ and p_B_ = 14.6 ± 0.9%. Except for residues 106, 210 and 212, the dispersion profiles of residues in the DNA-binding domain of the full-length RHR fitted to a single cluster with exchange parameters similar to those of the isolated DBD (*k*_ex_ = 1600 ± 100 s^−1^ and *p*_B_ = 5.1 ± 0.5%) (Supplementary Figure S5). The exchange parameters of Val210 and Leu212 were also similar to those seen for the isolated DBD, with *k*_ex_ = 660 ± 88 s^−1^ and *p*_B_ = 14 ± 2%. The dispersion curves for Leu106 in both the DBD and RHR did not fit to either cluster. The remarkable similarity in both the residues undergoing dispersion and in the fitted parameters strongly suggest that the same process is being observed in the isolated DBD and in the RHR homodimer. The residues that exhibit methyl ^13^C relaxation dispersion are located in a contiguous spine that extends from the DNA-binding loops to the opposite end of the DBD (Figure 4A and Supplementary Figure S6). Many of these residues are in the interface between the helices and the central β-sheet. The side chains of Leu67, Ile139, Val210 and Ile237 pack against the aromatic rings of Phe53 and Phe55, which are immediately adjacent to the DNA contact residues Arg54 and Arg56 (Figure 4B). Other DNA contact residues Y57 and T143 are also shown. These contacts provide a likely pathway by which the conformational fluctuations in the DNA binding loops, which result in exchange broadening of the backbone amide resonances of loop residues (Supplementary Figure S2B), are propagated to the core of the DBD.

At 300 K, the amide cross peaks of many residues in the DNA-binding loops are missing or strongly broadened but gain intensity as the temperature is lowered to 290K (Supplementary Figure S2). To confirm that these changes arise from changes in the kinetics of conformational fluctuations that lead to exchange broadening, we acquired methyl ^13^C relaxation dispersion data at 290 K (Supplementary Figure S7). The dispersion curves can be fit to a global process with *k*_ex_ = 480 ± 65 s^−1^ and *p*_B_ = 2.9 ± 0.4%, indicating a decrease in both the rate of exchange and in the population of the higher energy conformational state at the lower temperature. Taken together, the temperature dependent changes in the ^1^H–^15^N HSQC spectrum and in the ^13^C methyl relaxation dispersion profiles provide strong evidence for conformational fluctuations in the DNA-binding loops of unbound RHR that propagate into the core of the DBD.

### DNA binding by the p50 RHR homodimer

**Figure 5.**
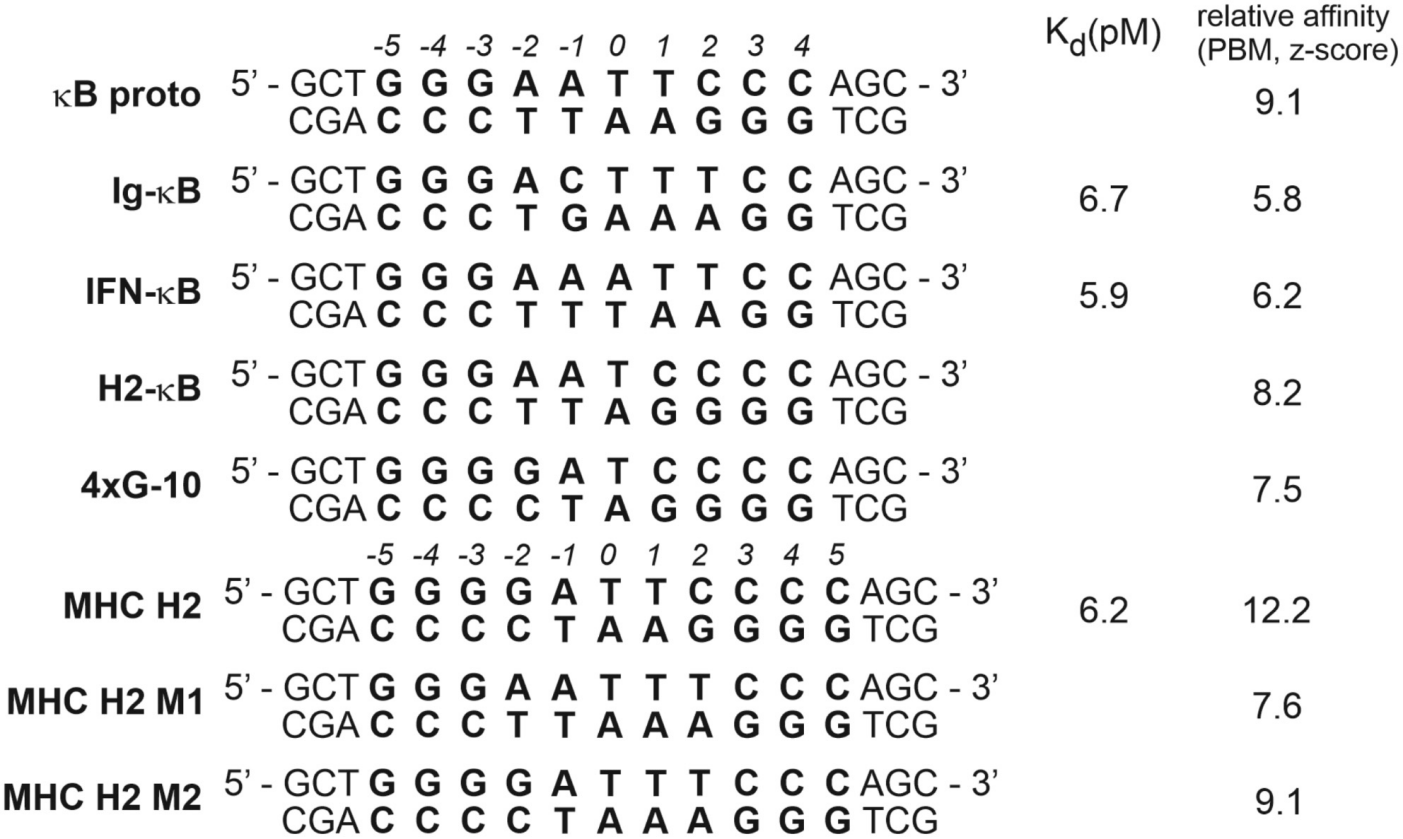
DNA duplexes. Ig-κB, IFN-κB, H2-κB and MHC H2 are naturally occurring κB sequences; κB proto is a consensus sequence (25); 4G-10 is a 10-base pair duplex with 4xG at both the 5′ and 3′ end. Mutants of the 11bp sequence MHC H2 are denoted M1 and M2. Published *K*_d_ values (8) for some of the naturally occurring κB sequences are shown to the right of the table. Values obtained from protein binding microarrays (*z*-score values) (24) are shown for each of the sequences. (Data from http://thebrain.bwh.harvard.edu/nfkb/Siggers_Chang_nfkb_pbm_dataset_12bp.txt). A larger number indicates tighter binding.

We next asked whether the presence of μs-ms motions in the DBD (and not in the DD) of the p50 RHR homodimer play a role in DNA binding and discrimination between κB DNA sites with different sequences. There is a fairly extensive literature (none of it using NMR) on the interaction of various members of the NF-κB family with a variety of DNA sequences. Interestingly, the literature indicates there is no difference in the (very high) affinity of p50 for these sites (8). We used NMR to investigate a series of DNA duplex sequences (Figure 5) for interaction with the p50 RHR homodimer. The dissociation constants (*K*_d_) for the natural κB sequences that we studied (IgκB, MHC H2, and IFN-κB) are in the pM range (Figure 5). Extensive comparison of κB sequences in protein binding microarrays (24) also showed largely comparable affinities (relative z-scores) for all of the sequences studied (Figure 5), although the affinity for MHC H2 appears to be the greatest. Measurement of NF-κB *K*_d_s by NMR is problematic, because the affinities are so high. *K*_d_ measurement by NMR is only possible when the interaction is in fast exchange, where the cross peaks shift from their positions in the free state towards their positions in the bound state as the ligand concentration is increased. For the DNA-p50RHR complexes, the affinities are all sufficiently high that the interaction is in slow exchange. This means that at intermediate concentrations of titrant, cross peaks of both free and bound states are observed, with intensities proportional only to the added ligand concentration. No information on *K*_d_ is available in these cases. The fact that all of the DNA oligonucleotides show similar behavior argues that the affinities are all comparable. When there are two cross peaks that appear on addition of (for example) κB proto, the observation that one cross peak is shifted to a much greater extent than the other led us to hypothesize that it represented an alternative mode of binding, but unfortunately the strength of this binding cannot be measured by an NMR titration, as explained above. The complexes of these DNA oligonucleotides with p50 RHR were characterized by native gel electrophoresis (Supplementary Figure S8). Each of these complexes shows a single band, consistent with the formation of a single complex between the p50 RHR homodimer and the various DNA oligonucleotides. Note that the mobility for the complexes of MHC H2, MHC H2 M1 and MHC H2 M2 is slightly higher than for the other DNA sequences. This is consistent with their length, 11 bp compared to 10 bp.

**Figure 6.**
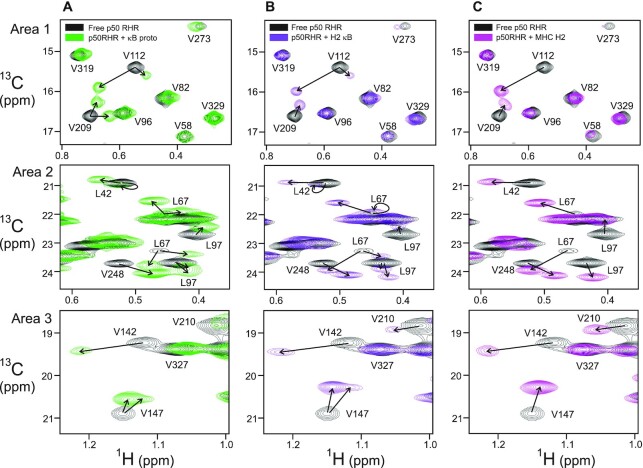
Overlay of three areas of the ^13^C methyl HMQC of p50 RHR free (black) and in complex with DNA duplexes (**A**) κB proto (green, (**B**) H2-κB (purple), and (**C**) MHC H2 (magenta). Assignments of cross peaks for the DNA complexes were inferred from the spectrum of the free protein and from a comparison of the spectra of all of the DNA complexes. The full overlaid spectra are shown in Supplementary Figure S9.

All of the oligonucleotide duplexes were designed with additional GCT/TCA tags at the 5′ and 3′ termini, to ensure duplex stability and avoid end effects. A p50 RHR:DNA complex was prepared with the same DNA oligonucleotide (κB proto, an optimized 10bp consensus κB sequence) as had been used in the 1995 X-ray crystal structure determined by Ghosh *et al.* (25). This complex gave a methyl HMQC spectrum where a number of peaks were shifted and several (e.g. L42, V58, V112, V147 and V209) were split into two component peaks (Figure 6A and Supplementary Figure S9A). This may result either from the presence of two conformations of the complex in solution, or from a single conformation with a different interaction of the DNA duplex with the two protomers of the homodimer. The crystal structure (25) showed the p50 homodimer bound symmetrically to the κB proto DNA, so any asymmetry in the complex in solution would imply a major difference from this structure. The intensity ratios for the split peaks in the spectrum of the κB proto complex (Figure 6A and Supplementary Figure S9A) indicate that at least two conformers with different populations are present. The relative intensities of the two conformer cross peaks differ between residues, for example, the peaks belonging to V209 are of comparable intensity, while those associated with V112 differ in intensity, a possible indication that more than two conformers are present. It is highly probable that the X-ray structure (25) was able to capture only the major conformation that we observe in our solution NMR studies. The positions of the residues showing split resonances in the spectrum of one or more of the DNA complexes are mapped on the structure of the p50 DBD in Supplementary Figure S9B.

To test whether the length and composition of the κB site in the DNA sequence influences whether there is more than one bound conformation, complexes were formed with three other DNA duplexes containing natural 10 bp κB sites: Ig-κB, IFN-κB and H2-κB (sequences shown in Figure 5). All of these complexes show heterogeneity in the NMR spectrum (an example is shown in Figure 6B and Supplementary Figure S9C). Not all of the resonances showing heterogeneity are the same for each complex, indicating that at least two magnetically distinct environments are sampled by these residues of the p50 RHR when bound to DNA.

**Figure 7.**
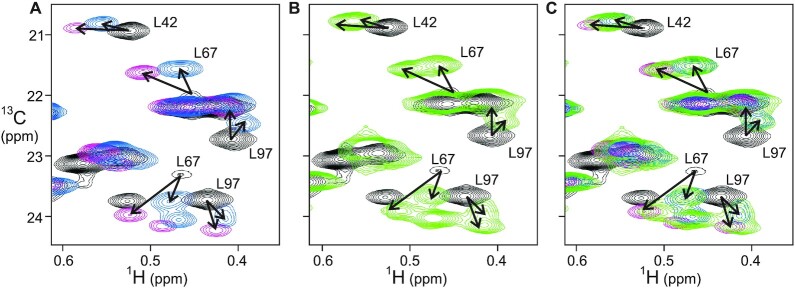
Overlay of portion of the methyl HMQC spectra of ILV-labeled p50 RHR homodimer (**A**) free (black), in the presence of MHC H2 (magenta), in the presence of M1 (blue) (**B**) free (black) and in the presence of M2 (green). (**C**) all four spectra. Complete overlaid spectra are shown in Supplementary Figure S10A.

Müller et al. determined a crystal structure of the p50 homodimer bound to a 19-mer oligonucleotide containing a longer κB recognition element, the 11bp MHC H2 DNA site (26). The methyl NMR spectrum of the ILV-labeled p50 RHR homodimer bound to the MHC H2 DNA duplex showed only one set of cross peaks (Figure 6C and Supplementary Figure S9D). The cross-peak positions observed for the MHC H2 κB site frequently correspond to one of the two split peaks observed for the complexes with the 10 bp duplex DNAs. We also observe that the intensity of several cross peaks that are weak in the spectrum of the free protein increases in the presence of the MHC H2 DNA duplex, suggesting that these regions of the protein have become stabilized in this complex. To identify the factors that promote a single conformation of p50 RHR homodimer in complex with the 11bp κB site, two mutant DNA sequences were prepared (sequences shown in Figure 5). The MHC H2 duplex is symmetric except for the T–A base pair at the middle position 0. MHC H2 Mutant 1 (M1) changed the identity of the nucleotides at positions ±2, from G–C to A–T base pairs. M1 reduces the GC content at each end of the κB site, which we hypothesized would change the local structure of the complex (κB sites are generally G-rich). Although this sequence resulted in a single p50 RHR bound conformation, the chemical shift perturbations were different from those of the MHC H2 DNA complex (Figure 7A, complete spectra shown in Supplementary Figure S10A), as might be expected if the structure had changed. The 11bp MHC H2 M1 remains terminally symmetric, as the replacement is made at both the + 2 and –2 positions in the duplex. To investigate whether breaking this symmetry would affect the interaction behavior of the duplex, we designed the MHC H2 mutant 2 (M2) DNA sequence. Splitting of cross peaks was observed in the ^13^C methyl HMQC spectrum of p50 RHR bound to this asymmetric 11bp duplex (Figure 7B, C, green), one set of which resembles the MHC H2 complex (magenta) and the other the M1 complex (blue) (Figure 7C). These results suggest that the binding of κB DNA sites to p50 RHR is modulated by both the length and the symmetry of the κB recognition site, and that the 5′ sequence of four consecutive guanines is important for the recognition process. To test whether the symmetrical 4xG sequence could operate in the context of the 10 bp κB site to give a single conformation of the complex, we added the 4xG-10 duplex (sequence shown in Figure 5) to p50 RHR. Two conformations of the complex were observed (Supplementary Figure S10B), indicating that the primary determinant for the formation of a single conformation of the p50 RHR is the presence of an 11 bp κB site.

### Dynamics of DNA-bound p50 RHR homodimer using methyl CPMG relaxation dispersion

**Figure 8.**
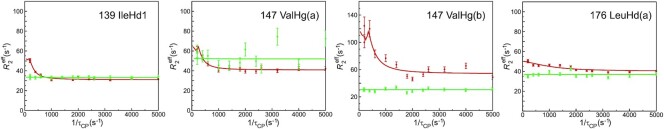
Overlaid representative methyl-selective ^13^C single quantum CPMG relaxation dispersion profiles collected at 18.8 T for selected residues of free p50 RHR (red; same data set as in Figure 3C) and p50 RHR:MHC H2 DNA complex (green). A complete set of all resolvable relaxation data profiles for the complex is shown in Supplementary Figure S11.

Evaluation of the effects of DNA binding on the dynamic behavior of the p50 RHR homodimer bound to DNA using methyl CPMG relaxation dispersion experiments was extremely difficult, due to the molecular size of the complex and its long-term instability at the high concentration necessary to obtain satisfactory signal in the CPMG experiment. We were able to prepare a single sample of the p50 RHR homodimer in complex with the MHC H2 DNA duplex at 250 μM, for which we obtained a single set of relaxation dispersion data over 3 days at 18.8 T. Precipitation of the complex after 3 days precluded the repeat of the measurement at 11.7 T. The data showed that many of the residues that showed dispersive behavior in the free form exhibited flat dispersion profiles in the DNA-bound p50 RHR complex (Figure 8, all resolved relaxation dispersion curves are shown in Supplementary Figure S11). In particular, the ^13^C methyl relaxation dispersion exhibited by residues 67, 97, 139, 147, 210, 212 and 237, which are located near the DNA binding loops, is abrogated upon binding of p50 RHR to MHC H2 DNA (Supplementary Figure S11B). These results clearly show that μs–ms timescale dynamics in the core and DNA-binding loops of the free p50 RHR homodimer are strongly damped by binding of DNA. In contrast, the methyl resonances of Ile104 and Val233 exhibit similar dispersion profiles in both free RHR and the DNA complex, showing that the exchange process experienced by these surface residues is unaffected by DNA binding.

## DISCUSSION

The p50 RHR homodimer recognizes a 5 bp long 5′-half site beginning with 5′-GGG; the 3xG sequence is essential for binding of p50 RHR with κB sites (27). Literature studies (27,28) suggest that the p50 RHR homodimer binds preferentially to an 11bp κB DNA composed of two 5 bp half sites separated by a central A:T. Available *K*_d_ values do not show differences in affinity of the various κB sites for (p50)_2_. An extensive protein binding microarray study (24) showed some variation in the apparent affinity of (p50)_2_ for the DNA sequences shown in Figure 5, with the MHC H2 sequence showing the greatest apparent affinity. Nevertheless, the relative values shown in Figure 5 do not seem well correlated with the presence of a single conformation in the NMR spectrum of the complex: the two κB sequences that show a single conformation (MHC H2 and MHC M1) have different apparent affinities (*z*-scores of 12.2 and 7.6), while the *z*-scores of those sequences that show more than one conformation range from 5.8 to 9.1. Measured affinity does not seem to be the determinant for the observation of a single conformation.

Variations in κB site sequences can result in quite different transcriptional outcomes (8). Our NMR studies of the binding of different κB DNA sequences give atomic-level insights into the specific requirements for binding to the p50 homodimer. We show that both the length of the κB DNA site and the composition and symmetry of the oligonucleotide influence whether the complex shows a single conformation or multiple local conformations. An 11 bp κB site is a necessary condition for the formation of a single conformation in the DNA complex. All of the 10bp sites showed the presence of two or more local conformations in the p50 RHR complex. In the context of the 11 bp site, the presence of a 3- or 4-guanine sequence at each of the 5′ ends of the duplex is a determining factor. Both the MHC H2 site and the M1 mutant are terminally symmetrical 11bp sites, with 5′ 4xG and 3xG sequences respectively, and both show the presence of a single bound conformation of p50 RHR. The chemical shift changes for the MHC H2 site are greater than those for the M1 site, which could be an indication that the terminally symmetrical 4xG site has a greater affinity for p50 RHR than the 3xG site, but certainly indicates a difference in the local structure surrounding these methyl groups. By contrast, the non-symmetrical M2 mutant, which contains a 4xG site at one 5′ end and a 3xG site at the other, shows two conformations in the p50 RHR complex. Further, the positions of the M2 complex cross peaks correspond for several residues to the cross-peak positions of the MHC H2 complex and the M1 complex. We infer that p50 RHR is binding to the M2 site in two orientations, one corresponding to the 4xG site found in MHC H2 and the other corresponding to the 3xG site found in M1. The behavior of the M2 mutant thus provides insight into the structural basis for the formation of multiple complexes with p50 RHR.

An interesting variant is seen in the behavior of the 10 bp H2-κB complex, which contains one strand with 3xG and one with 4xG. While this complex also shows two conformations, it appears that the bound conformation differs in the H2-κB complex from that in the κB proto complex, as indicated by the difference in the shift of the cross peaks from the free form (see, e.g., Area 2 in Figure 6). This observation may implicate a more definitive role for the 4xG 5′ sequences, as suggested previously (27). Nevertheless, the 4xG-10 sequence containing two 5′ 4xG sequences showed two conformations of the p50 RHR complex, indicating that the 11bp length is the primary determinant of the specificity for a single conformation. These observations imply that the presence of an additional base pair in the κB site gives extra stability and specificity to the structure of the complex, allowing it to sample only one complex conformation.

The complex with the 11 bp κB shows increased intensity for several methyl cross peaks (for example, for residues Leu67, Ile139, Val210 and Ile237) that are weak in the spectrum of the free protein. This is an indication that the presence of the 11bp DNA has damped the local motions in this region, which were detected in the free protein by the relaxation dispersion experiments. Local motion and multiple local conformations can result in the broadening and disappearance of NMR resonances. In the structure of the complex, the side chains of these residues form a hydrophobic cluster with the aromatic rings of F53 and F55 (Figure 4). The adjacent arginines, R54 and R56, recognize the guanines at the –4 and –3 positions. Another residue that forms part of the hydrophobic cluster, L97, shows significant chemical shift changes upon complex formation (Figures 6 and 7). The chemical shift changes upon DNA binding show that conformational changes are propagated from the DNA-binding loops through the hydrophobic core, possibly indicating a repacking of the core together with the damping of the dynamics observed in the free protein. The chemical shift changes in these hydrophobic clusters can also explain the observations for the mutants M1 and M2 of MHC H2: they are likely due to changes in the position of the two Phe rings when the adjacent Arg side chains interact with the G at the -3 position. In MHC H2 itself, this G is adjacent to the G at position -2 at both ends of the site. In the M1 mutant, it is adjacent to an A at position –2, again at both ends of the site. The methyl peaks in the DNA loop shift to a different position in M1 due to the presence of A rather than G. By contrast, in M2, two environments are sampled, one with G at position –2 and one with A at –2, thus recapitulating the cross peak positions of MHC H2 and M1 (Figure 7).

These results highlight the importance of guanine-rich DNA sequences for modulating the formation of complexes between DNA κB sites and p50 RHR. The differences in the interaction of the p50 RHR homodimer with guanine-rich sequences of various lengths and compositions has implications for the specificity of the cellular response to pathogens. For example, NF-κB p50 has been found to regulate antimicrobial immunity through binding with DNA sequences containing κB and interferon response elements (IRF) sites (7). It was observed that a single half-site containing three or four guanines is sufficient for making strong contacts with the first p50 monomer enabling high affinity binding, whilst the other monomer can accommodate a non-consensus sequence.

**Figure 9.**
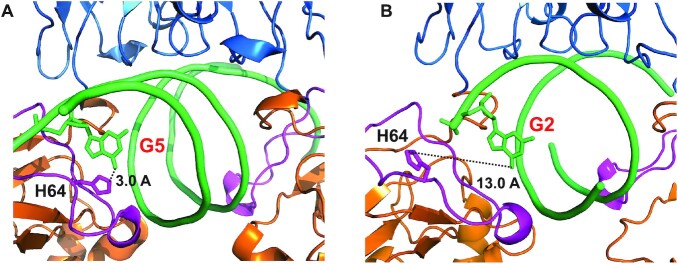
Portion of the X-ray crystal of p50 RHR homodimer complexed with (**A**) the 19-mer oligonucleotide (AGAT**G**GGGAATCCCCTAGA; G5 in red) containing the 11bp κB site MHC H2 (PDB 1SVC) (26) and (**B**) the 11-mer oligonucleotide (T**G**GGAATTCCC; G2 in red) containing the 10 bp κB site κB proto (PDB 1NFK) (25). The DNA backbone is shown in green, with the nucleotide G5 on one strand shown as sticks. The p50 RHR backbone is colored orange (DBD), blue (DD) and magenta (loop AB of the DBD), as in Figure 1B. The side chain of H64 in one DBD is shown in magenta sticks, with the distance between the O6 of G5 and the Cϵ of H64 shown as 3.0 Å in (A) and 13.0 Å in (B).

Previous studies have shown that the binding of p50 RHR homodimer with sequence-specific DNAs results in limited digestion with trypsin, chymotrypsin, or proteinase K (29,30). In the absence of DNA, the entire p50 protein was degraded by trypsin. Binding of p50 RHR to the high affinity MHC H2 κB site provides complete protection against cleavage by trypsin on the C-terminal side of the K74 residue, but the extent of protection was less when p50 was bound to the Ig-κB sequence (sequences shown in Figure 5). It was proposed that DNA binding masks the trypsin cleavage site by causing a conformational change in the p50 structure (30), but the nature of this conformational change is obscure, since the affinities of the different variant κB sequences for (p50)_2_ are so similar (8,24). Similarly, the sensitivity of p50-DNA complexes towards cleavage by chymotrypsin depends on the precise sequence of the bound DNA (29). The conformational change implied by the protease digestion results may rather be a consequence of the differences in the dynamic behavior of the p50 RHR that we observe between the free protein and the DNA complexes. Our results, together with the Müller crystal structure (PDB: 1SVC) (26) of the 11bp DNA complex, suggest that the longer DNA κB site containing the 4xG 5′ sequence causes the rigidification of part of the p50 N-terminal domain AB loop, which contains three lysine residues, due to the formation of additional contacts with backbone phosphates outside the 11 bp MHC H2 κB site and between the G(–5) base and the side chain of His64 (Figure 9A). On the other hand, a 10 bp κB sequence such as κB proto, even one containing 4xG at either end, was not able to make stable contact with His64 due to its shortened length (Figure 9B). As a result, the DNA recognition loop region is relatively flexible, as indicated by the presence of two conformations in the NMR spectrum, and could thus be penetrated by proteases.

**Figure 10.**
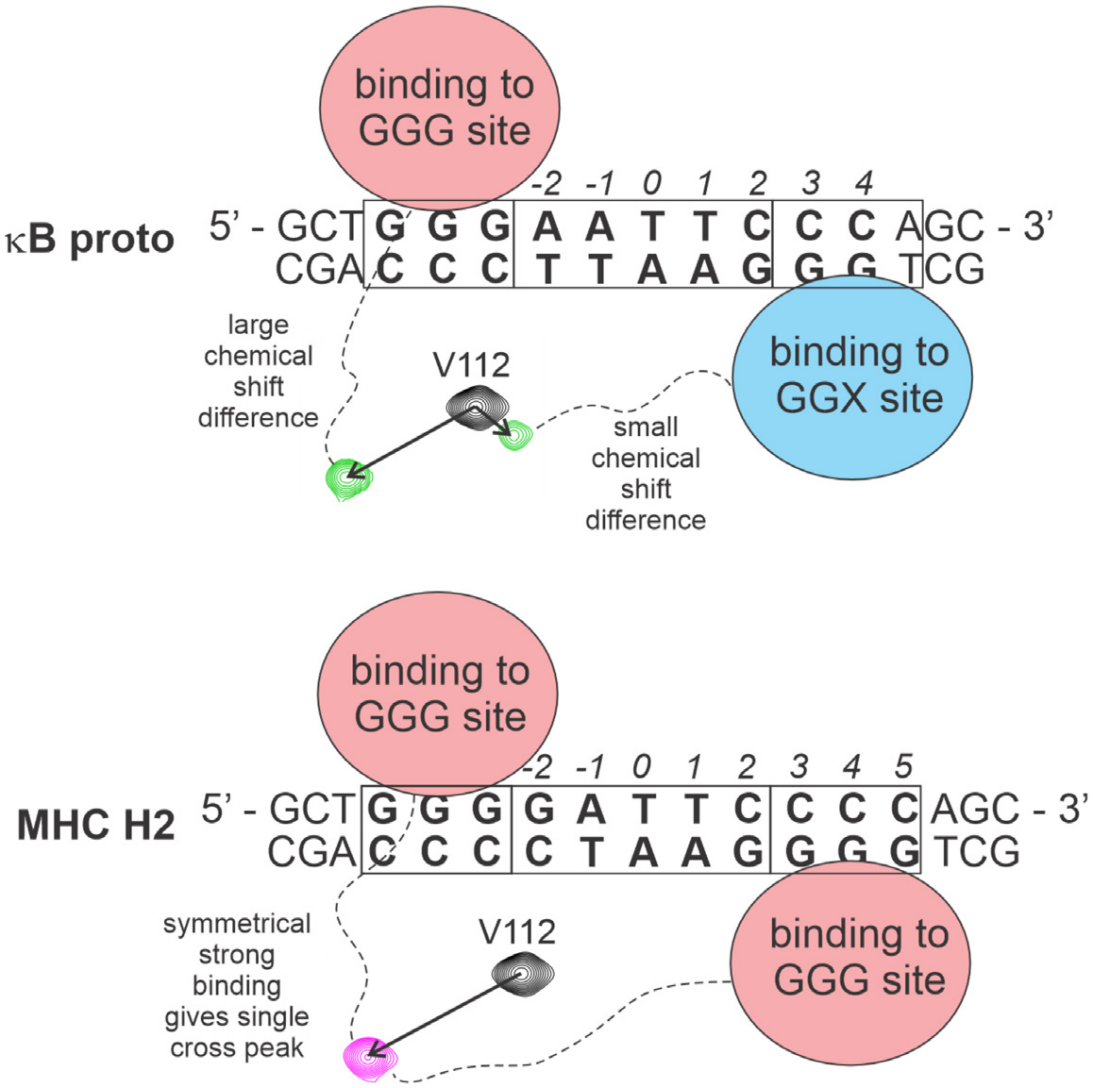
Schematic diagram showing the source of the doubled cross peaks for p50 RHR in complex with 10 bp κB sequences. A sequence such as κB proto contains the 5′ GGG sequence required for strong binding to a DBD of the p50 RHR homodimer. However, given the half-site spacing of five nucleotides, the binding of the second DBD will be to the non-optimal GGT sequence. For the MHC H2 sequence, optimal binding can occur for both DBDs. The assignment of the two cross peaks in the spectrum of V112 in the κB proto complex, and of the single cross peak in the MHC H2 complex, is illustrated using portions of the spectra from Figure 6.

We conclude that the 10bp κB site, despite its presence in several natural sequences, is simply too short for uniform symmetric binding in the (p50)_2_ complex. This is illustrated in Figure 10. The positions of the methyl cross peaks in the presence of the various κB sequences is correlated with the local structure of the DBD–DNA binding site. Cross peaks in the same position imply that the local structure at the interface is the same. When the p50 RHR homodimer binds to a κB sequence, one of the DBDs of the dimer binds to the GGG (in κB proto) or GGGG (in H2 κB or MHC H2) sequences in the same conformation as when bound to M1 or M2, as shown, for example, by the positions of the L67 cross peaks in Figures 6 and 7. The half-site separation for NF-κB consists of 5 bp between the two GG motifs that bind R54 and R56 of the recognition loop. Assuming that the half-site spacing is maintained, the second DBD of the p50 RHR homodimer will bind in a different manner to the 3′ terminal sequence (i.e. the 5′ end of the bottom strand of the DNA duplex) of the 10 bp κB site, one of which is an AT base pair in our κB proto oligonucleotide. The interaction at this second site may be weaker than at the other site, indicated by the broad and weakly-shifted second peaks in the methyl spectra, but we cannot obtain quantitative information on comparative affinities from our NMR experiments, due to the circumstance, mentioned previously, that the complexes are in slow exchange on the NMR time scale. The preferred site is GGG, which is available at both ends of the 11bp sequences.

Polypeptide chain dynamics clearly play a prominent role in the free DBD, both in isolation and as part of the p50 RHR homodimer. The broadening of the backbone NH resonances in the DNA-binding loops of the p50 DBD is a strong indication that this region of the protein is fluctuating on a time scale that is intermediate on the NMR chemical shift time scale. Several of the cross peaks that are missing from the ^1^H–^15^N HSQC spectrum at 300 K are observed at 290 K, an indication that the backbone motions are approaching slow-exchange timescales, but the degree of exchange broadening in the backbone NH resonances, particularly in the areas of greatest interest, the DNA-binding loops, remains too large for relaxation dispersion measurements. The ^13^C relaxation dispersion data for the methyl groups of Ile (δ), Val and Leu provide evidence for μs-ms time scale motions that extend beyond the DNA-binding loops into the hydrophobic core. For both the isolated DBD and the RHR homodimer, the residues that exhibit ^13^C methyl relaxation dispersion are distributed along a hydrophobic spine that extends from the DNA-binding loops along the interface between the helices and the central β-sheet. The μs-ms time scale conformational fluctuations in this spine are coupled to those in the DNA-binding loops and are damped upon binding of DNA.

While much of the p50 DBD shows evidence of μs-ms motions in the free state, the fluctuations that lead to broadening of backbone amides and methyls in the contact loops are concentrated in the vicinity of the DNA binding site. Flexibility and dynamic exchange between two or more conformational states in the unbound DBD would be expected to facilitate target search and enhance specificity by allowing the DNA-binding loops to enter the major groove of the duplex to form optimal interactions with the cognate recognition elements but not with non-specific sites on the DNA. Upon binding to a cognate 11bp MHC H2 DNA target site, the μs-ms conformational fluctuations of the p50 DBD are quenched (Figure 8). Binding to a sub-optimal 10 bp recognition element leads to two or more distinctive bound conformations of p50 RHR, due to the half-site spacing: when the spacing is too short, one DBD forms optimal interactions (indicated by the similarity in the cross peak positions), while many methyl cross peaks of residues in the other DBD are weak and, for some residues, are close in chemical shift to the free protein (see, for example, Val112, Leu67 and Leu97 in Figure 6). The complex with a 10 bp site gives greater cross peak intensity than that with a non-cognate DNA sequence, but retains some degree of heterogeneity and is likely to be more labile than the complex with a fully-specific 11bp site. This characteristic speaks to the role of the p50 homodimer in repression of transcription (6,7), by masking sites from interaction with promoter NF-κBs such as the p50–p65 heterodimer.

It is possible that DNA bending contributes to binding, but we have seen no evidence of this. All of our measurements are made on the protein portion of the complex – our experiments give no information on the state of the DNA in solution. Our only insight into the structure of the DNA within the complex comes from crystal structures of NF-κB complexes with DNA. The 1NFK structure (25) is described as ‘an undistorted B-DNA helix’, while the 1SVC structure (26), which contains a central A-A mismatch that is not present in our oligonucleotides, is reported to be bent, with an increased overall twist and a very deep major groove. These authors note that ‘because the continuous duplex is only 11 bp long, the grooves are open at either end, and it is not possible to make strong conclusions about the actual overall bend’. Our oligonucleotides contain neither the central mismatch of the 1SVC DNA nor the open ends. The oligonucleotides MHC H2, MHC H2 M1 and MHC H2 M2 are all base-paired 11-nucleotide duplexes. If the bend noted in the 1SVC crystal structure were to be an important determining factor in the presence of two local conformations, we should expect that these three oligonucleotides should show the same behavior, yet we observe one conformation for MHC H2 and MHC H2 M1 and two conformations for MHC H2 M2. This observation argues against bending of the DNA as an explanation for the two local conformations that are observed in some, but not all, of the κB sequences we examined.

What is the significance of the observation of two local conformations of the p50 RHR homodimer in the presence of some, but not all, κB sequences? An examination of the transcriptional preferences of NF-κB dimers p50–p65, (p50)_2_ and (p65)_2_ revealed different activation mechanisms (8). Transcriptional activation by the heterodimer p50-p65 is mediated by the C-terminal activation domain (CTAD) of p65, which can also mediate transcriptional activity of the p65 homodimer. The p50 homodimer lacks a CTAD but is capable of transcriptional activation of some κB sequences but not others. The sequences tested (8) included the same Ig-κB, IFN-κB and MHC H2 sequences that we report here. All three sequences showed comparable binding affinity to (p50)_2_ (Figure 5), but only the MHC H2 sequence shows significant transcriptional activity. Interestingly, the MHC H2-(p50)_2_ complex is the only one of the three that is chymotrypsin-resistant (8). It appears that the local conformational heterogeneity that we observe for the complexes of Ig-κB and IFN-κB confers sufficient flexibility and solvent accessibility on (p50)_2_ for cleavage by chymotrypsin, and by analogy, sufficiently perturbs these complexes such that transcriptional activity is no longer promoted. By contrast, the transcriptionally active MHC H2 complex is chymotrypsin-resistant and also shows a single conformation in the methyl NMR spectrum. These observations support the proposal that DNA binding by MHC H2 results in a conformational change from a dynamic state to a more rigid structure (30). Local polypeptide chain dynamics in this case, rather than absolute measurable affinity, is likely the determining factor in the transcriptional activity of the p50 homodimer. It is tempting to speculate that the conformational heterogeneity on the shorter κB sites, with sub-optimal interactions of one subunit with DNA, exposes one of the p50 molecules to chymotrypsin proteolysis. On the longer symmetric H2 site, both subunits make optimal interactions with DNA and are protected from cleavage.

Protein conformational transitions have frequently been invoked to account for the ability of transcription factors and other sequence-specific DNA-binding proteins to search rapidly for their targets through transient interactions with non-specific DNA sequences yet form stable complexes at their cognate recognition elements. A large body of structural and thermodynamic data shows that conformational fluctuations in the unbound state and local protein folding processes play a key role in sequence-specific DNA recognition (31,32). Theoretical considerations suggest that protein conformational fluctuations between nonspecific and specific binding modes are essential to allow rapid scanning of nonspecific DNA and tight binding at specific sites, thus resolving the so-called speed-stability paradox (33–36). Our current work provides experimental support for these models, revealing millisecond time scale conformational fluctuations in the DNA binding loops and other regions of the p50 RHR DBD that are fully quenched upon binding to an optimal DNA site. Similar observations were made for the lac repressor headpiece, which exhibits extensive μs-ms motions in the free state that are quenched upon binding to specific but not to non-specific DNA sequences (32).

In conclusion, our current solution NMR data suggest the presence of conformational plasticity in the free p50 RHR homodimer that could be relevant for DNA complex formation and the selection of specific DNA sites by various NF-κBs. Our dissection of the atomic-level factors that promote fully-specific interaction of p50 RHR with 11bp DNA sites and slightly less-specific interaction (with 2 or more bound conformations) with 10bp or unsymmetric sites also provides insights into possible mechanisms for the varying roles of different members of the NF-κB family in transcription.

## DATA AVAILABILITY

The fitting program Glove is available on the NMRBox platform (www.nmrbox.org).

All NMR resonance assignments and crystal structure coordinates have been previously published and uploaded to the relevant databases.

## Supplementary Material

gkac535_Supplemental_File

## Notes

Present address: Amrinder Singh, Department of Structural Biology, St. Jude Children's Research Hospital, 262 Danny Thomas Place, Memphis, TN 38105-3678, USA.
