# Supplementary material for: Structural and dynamic studies of DNA recognition by NF-κB p50 RHR homodimer using methyl NMR spectroscopy
Source: Nucleic Acids Res. 2022 Jun 24;50(12):7147–60. doi: 10.1093/nar/gkac535 (PMC9262625; doi:10.1093/nar/gkac535)
Supplement: gkac535_Supplemental_File [file gkac535_supplemental_file.pdf]

Supplementary Information

for

Structural and dynamics studies of DNA recognition by NF-κB p50 RHR homodimer using methyl NMR spectroscopy

Amrinder Singh, Maria A. Martinez-Yamout, Peter E. Wright and H. Jane Dyson*

Department of Integrative Structural and Computational Biology, Scripps Research, 10550 North Torrey Pines Road, La Jolla CA 92037, USA

*Corresponding Author: email:dyson@scripps.edu

ORCID: HJD 0000-0001-6855-3398

PEW: 0000-0002-1368-0223

MAM-Y: 0000-0003-4376-437X

AS: 0000-0002-1677-337X

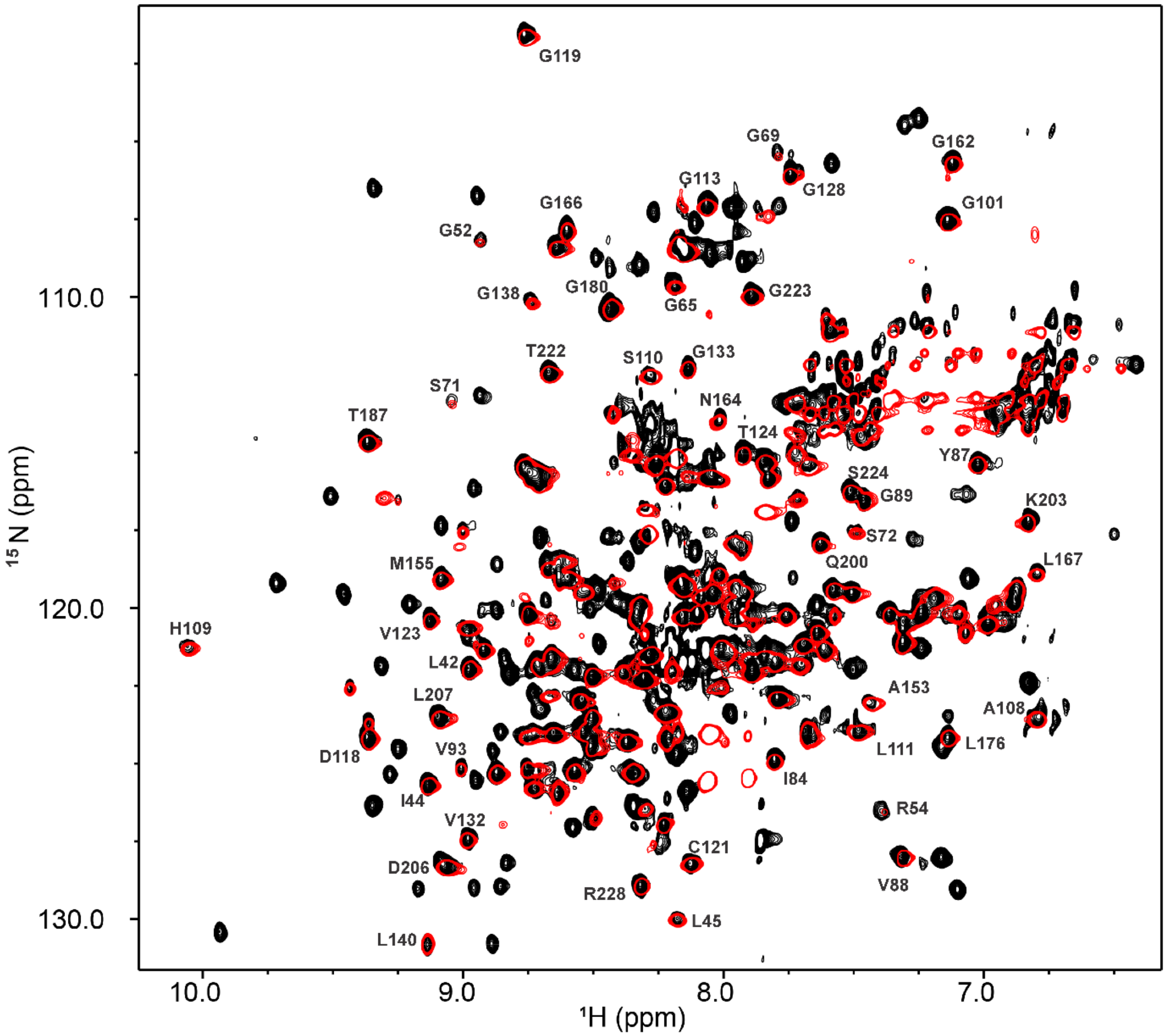

Figure S1A. Overlaid NMR spectra: ^{15}N TROSY of p50 RHR homodimer (black) and ^{15}N TROSY of p50 DNA binding domain (DBD) (red). Selected resonances from p50 DBD are marked on the spectrum. Spectra acquired at 900 MHz, 300K.

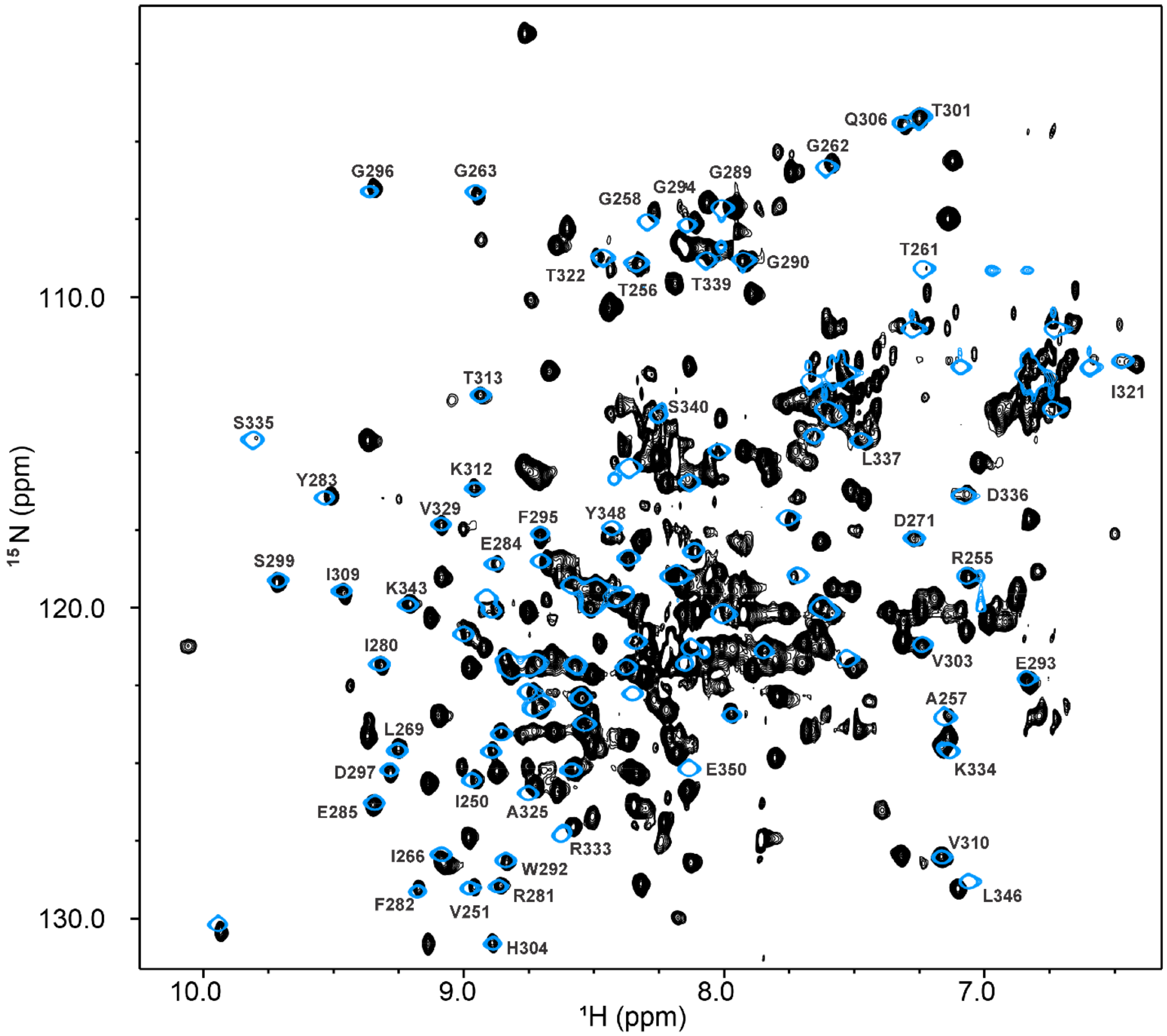

Figure S1B. Overlaid NMR spectra: ^{15}N TROSY of p50 RHR homodimer (black) with ^{15}N HSQC spectrum of p50 DD (dimerization domain) (blue). Resonances from p50 DD are marked on the spectrum. Spectra acquired at 900 MHz, 300K.

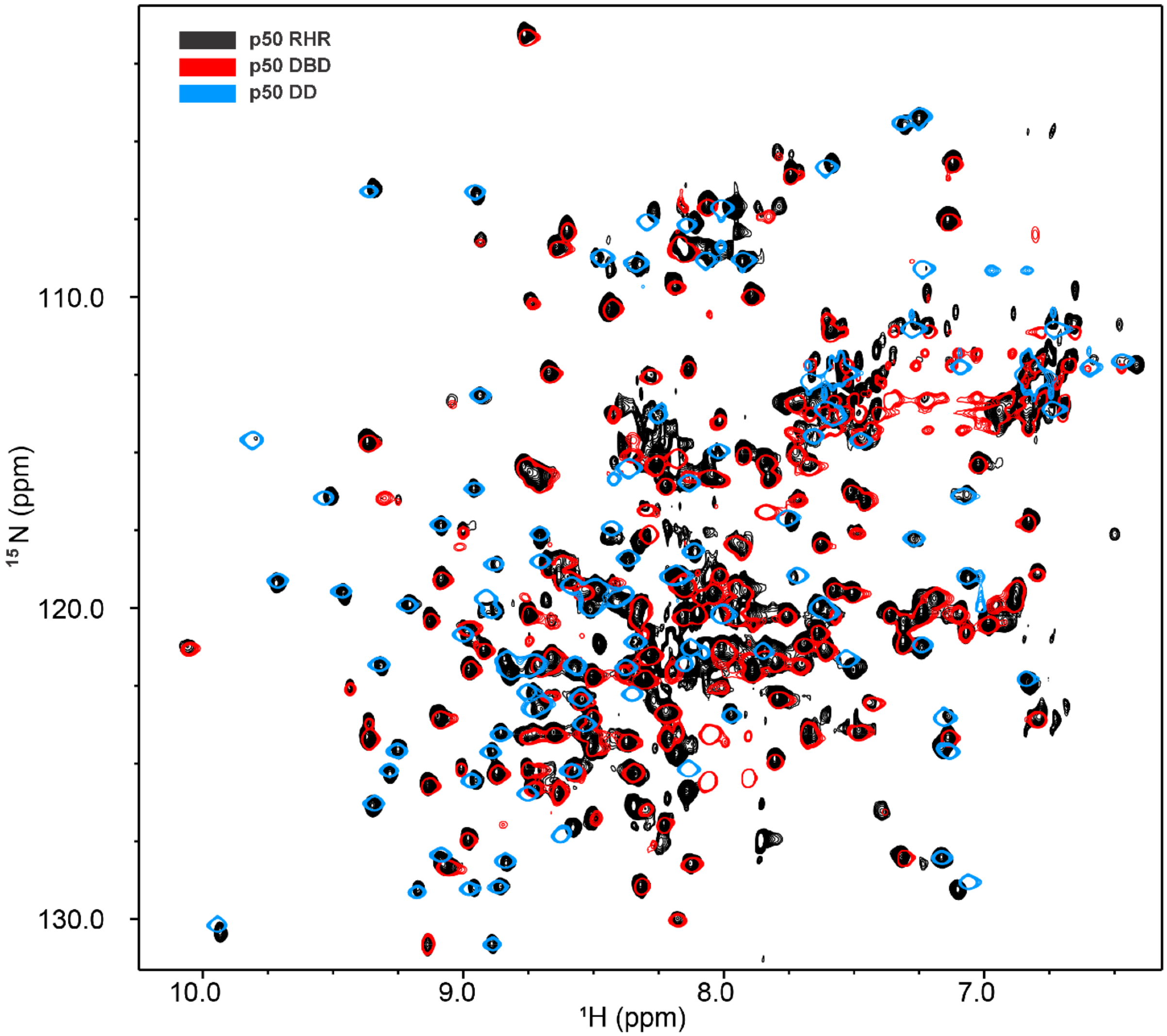

Figure S1C. Overlaid NMR spectra: ^{15}N TROSY of p50 RHR homodimer (black), ^{15}N TROSY of p50 DNA binding domain (DBD) (red) and ^{15}N HSQC of p50 dimerization domain (DD) (blue). Spectra acquired at 900 MHz, 300K.

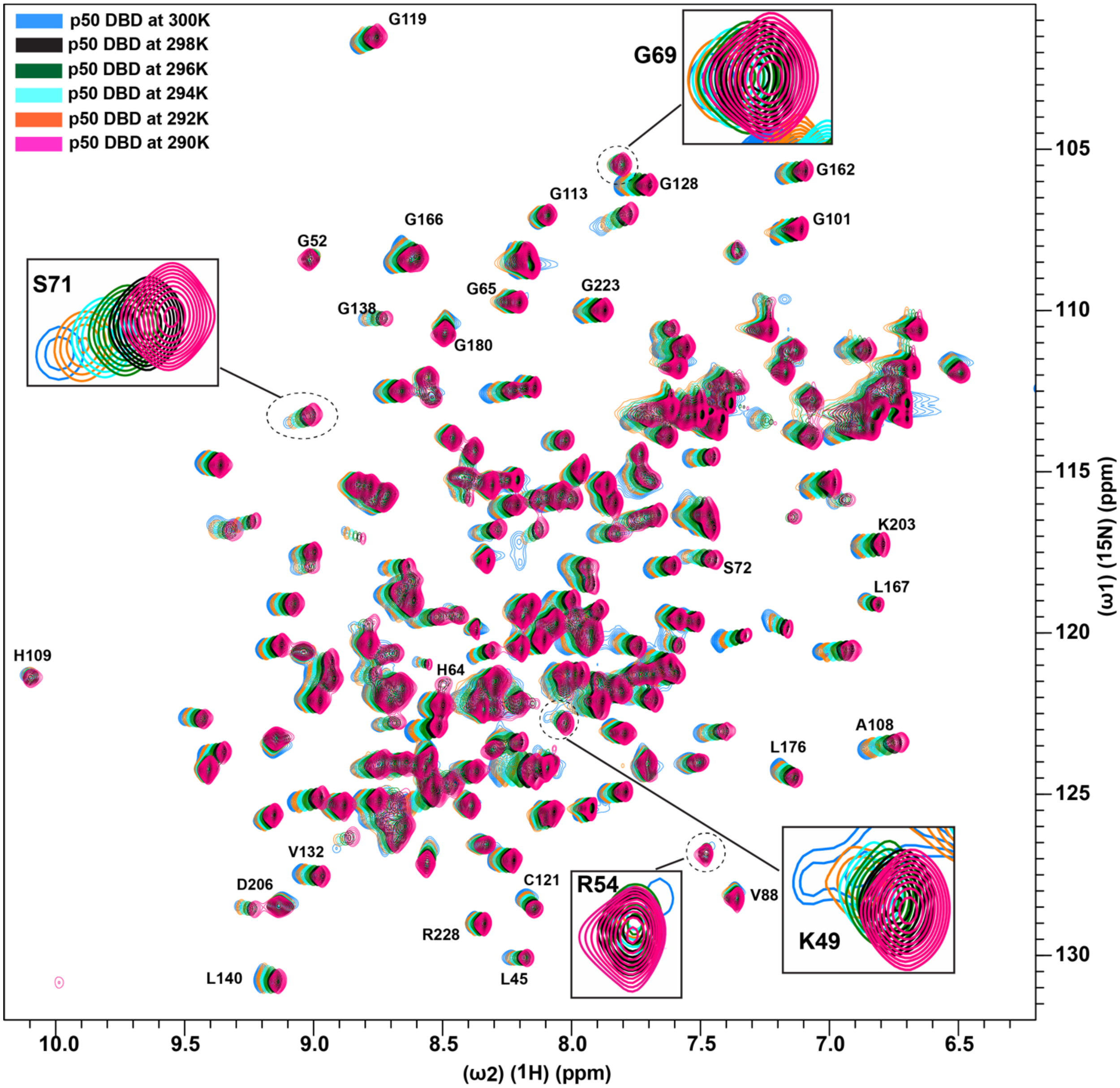

Figure S2A. Temperature dependence of ^{15}N HSQC of p50 DBD.

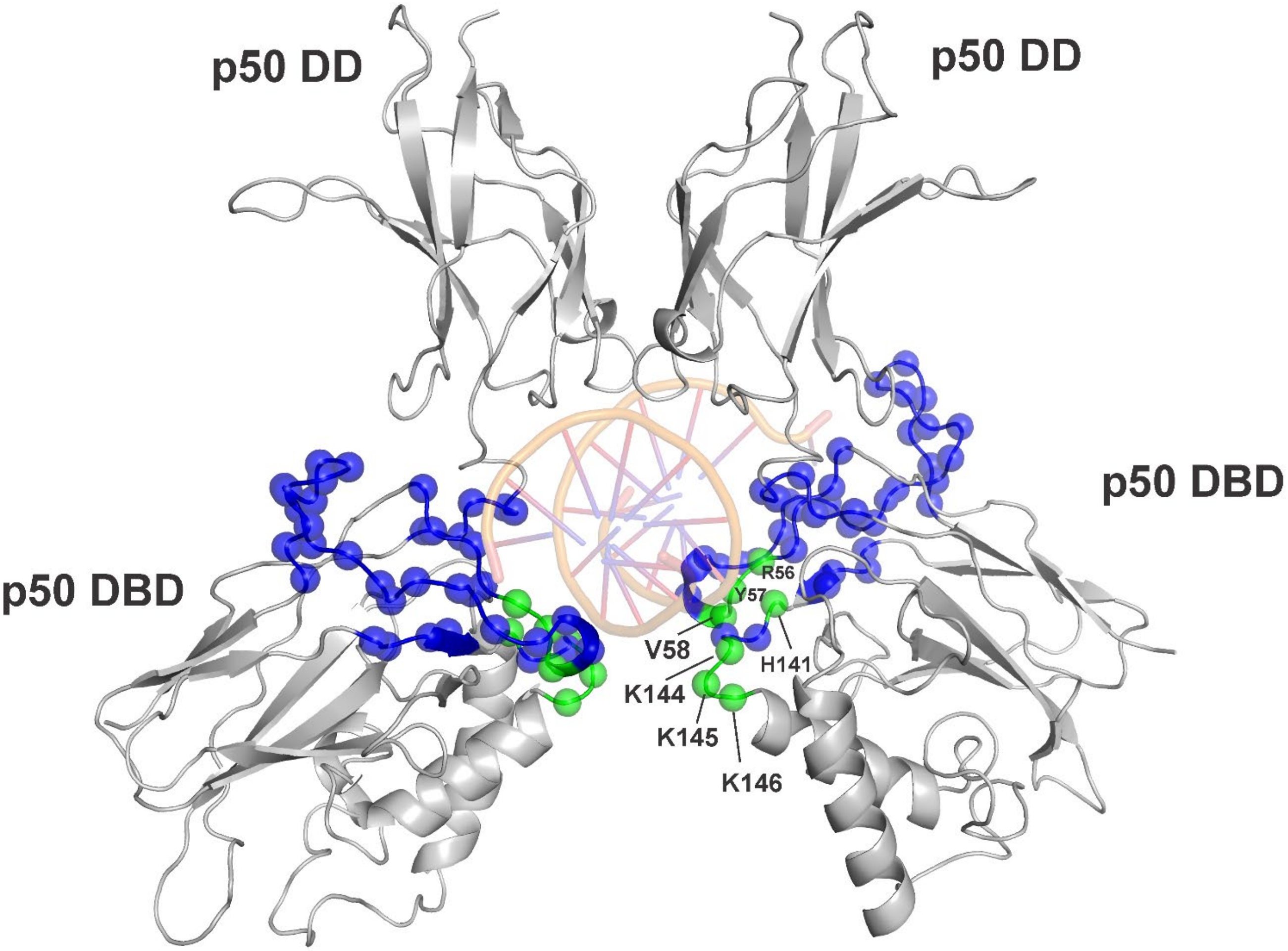

Figure S2B. Colored spheres represent the Cα of residues with resonances broadened or missing from the ^{1}H-^{15}N HSQC spectrum of free p50 DBD at 300K (blue) and at both 300K and 290K (green), mapped onto the structure of the DBDs in the p50 RHR homodimer DNA complex (pdb 1NFK). The DNA is not present, but is included to show its position.

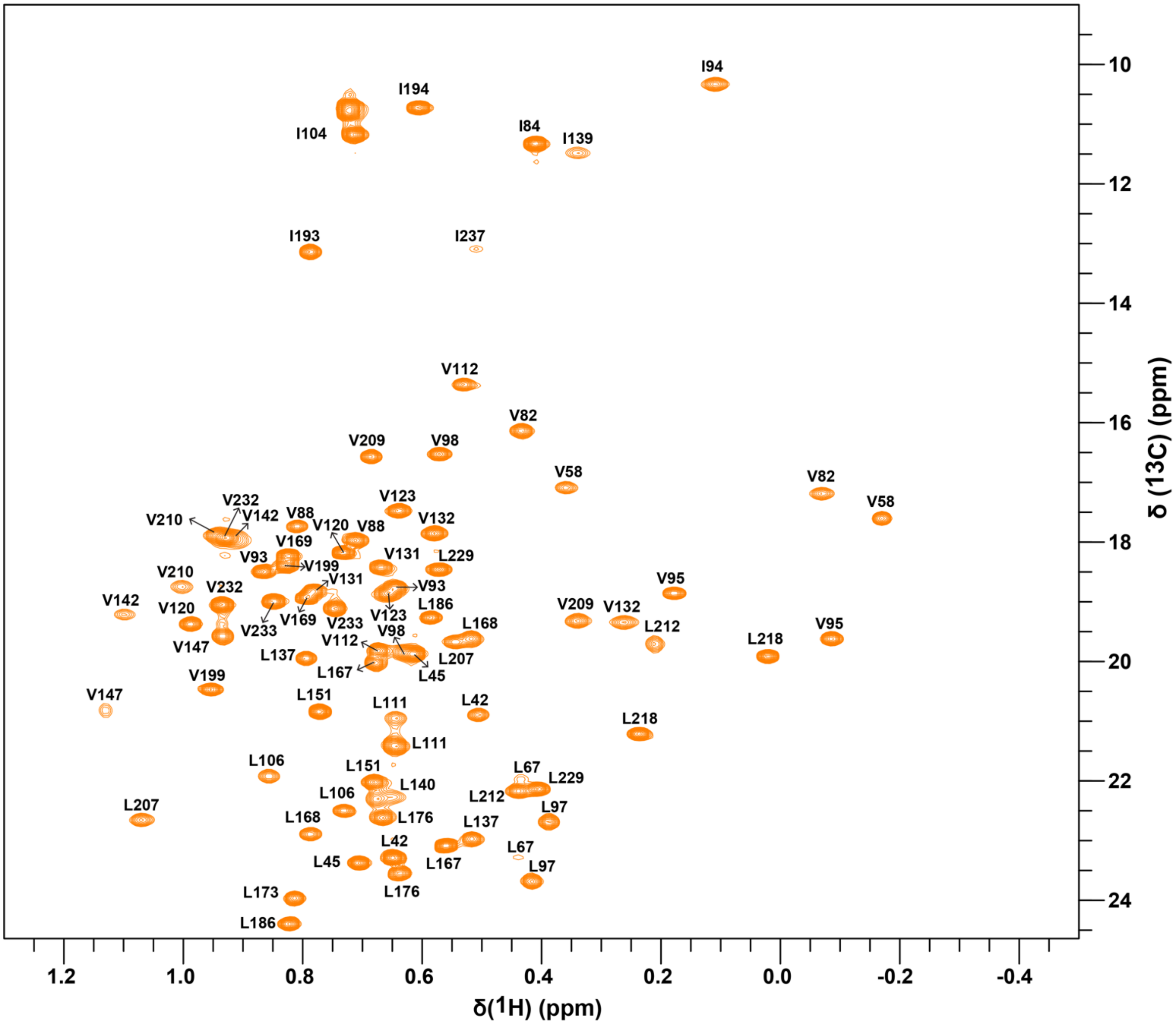

Figure S3A. ^{13}C methyl HMQC of p50 DBD collected at 300K, pH 6.8, 800 MHz spectrometer.

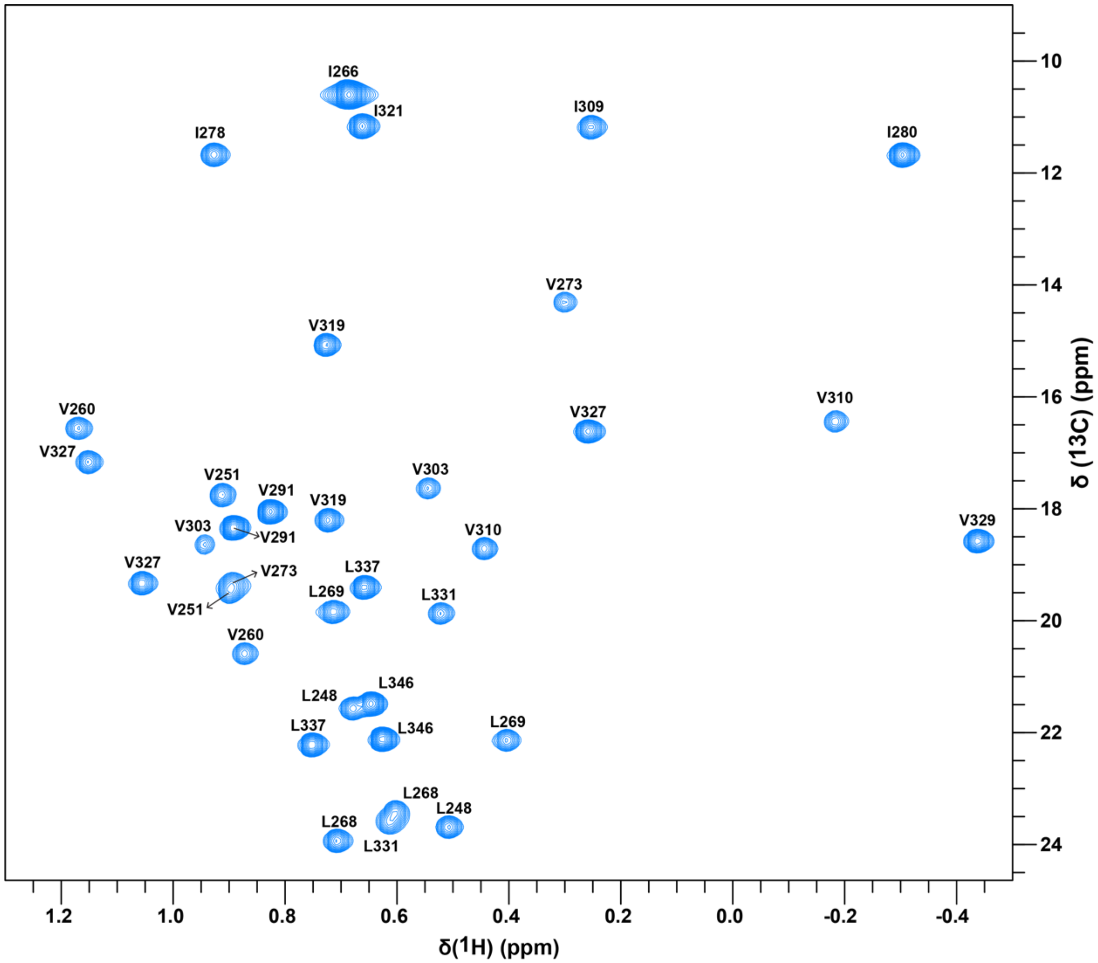

Figure S3B. ^{13}C methyl HMQC of (dimeric) p50 DD collected at 300K, pH 6.8, 800 MHz spectrometer.

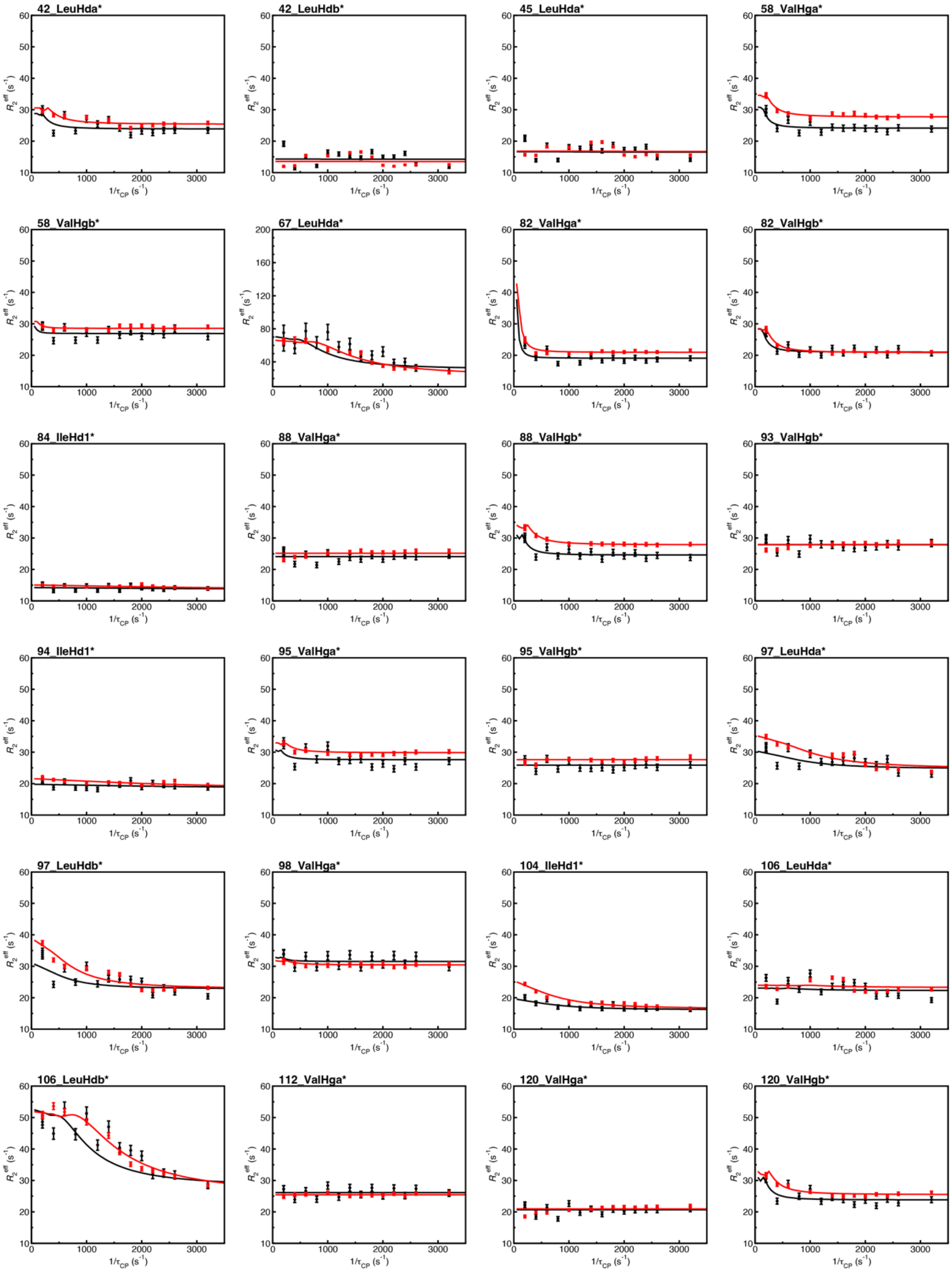
42_LeuHda*
42_LeuHdb*
45_LeuHda*
58_ValHga*
58_ValHgb*
67_LeuHda*
82_ValHga*
82_ValHgb*
84_IleHd1*
88_ValHga*
88_ValHgb*
93_ValHgb*
94_IleHd1*
95_ValHga*
95_ValHgb*
97_LeuHda*
97_LeuHdb*
98_ValHga*
104_IleHd1*
106_LeuHda*
106_LeuHdb*
112_ValHga*
120_ValHga*
120_ValHgb*
R_2^{eff} (s^{-1})
$1/\tau_{CP}$ (s^{-1})

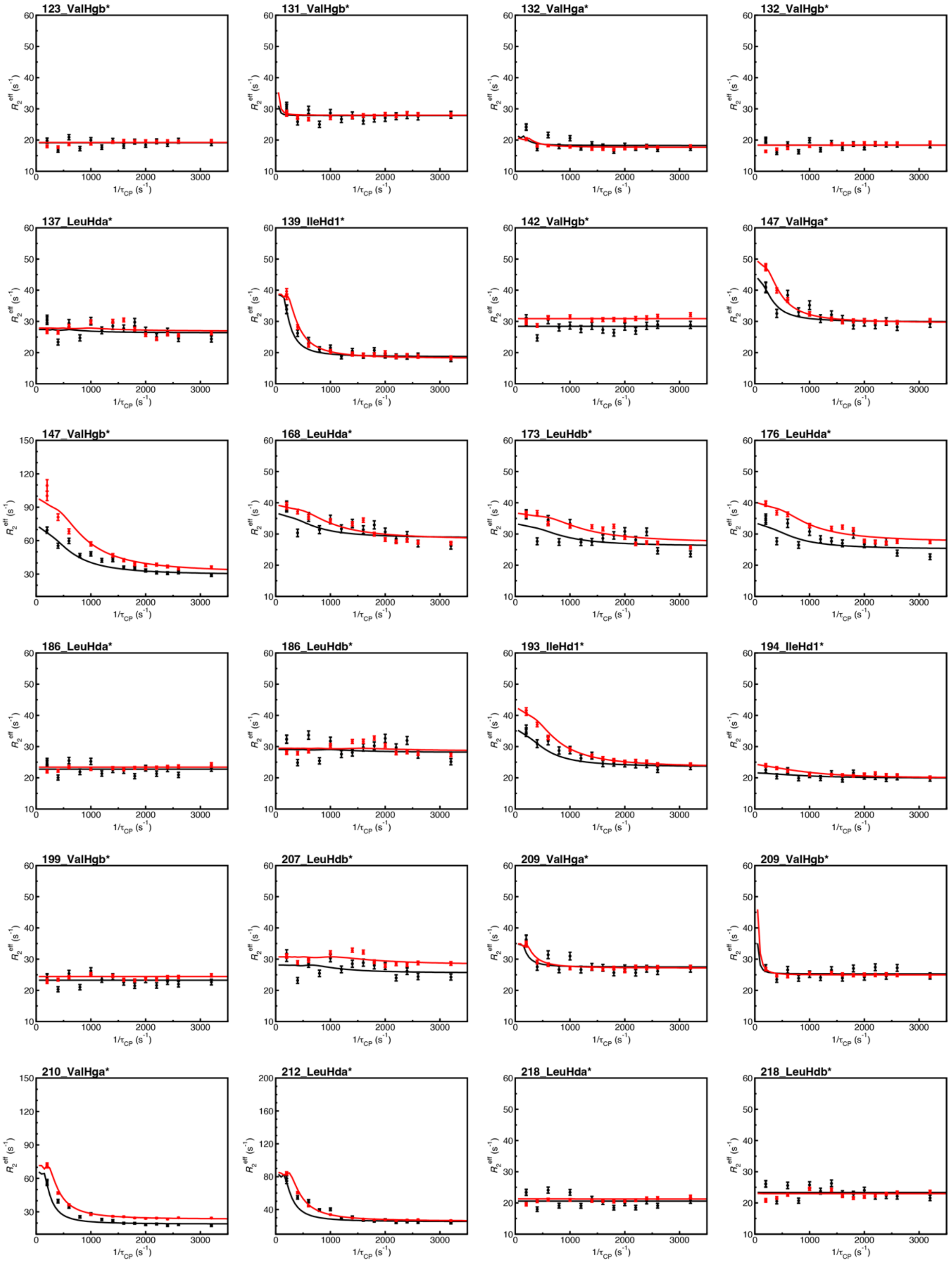
123_ValHgb*
131_ValHgb*
132_ValHga*
132_ValHgb*
137_LeuHda*
139_IleHd1*
142_ValHgb*
147_ValHga*
147_ValHgb*
168_LeuHda*
173_LeuHdb*
176_LeuHda*
186_LeuHda*
186_LeuHdb*
193_IleHd1*
194_IleHd1*
199_ValHgb*
207_LeuHdb*
209_ValHga*
209_ValHgb*
210_ValHga*
212_LeuHda*
218_LeuHda*
218_LeuHdb*
R_2^{eff} (s^{-1})
$1/\tau_{CP}$ (s^{-1})

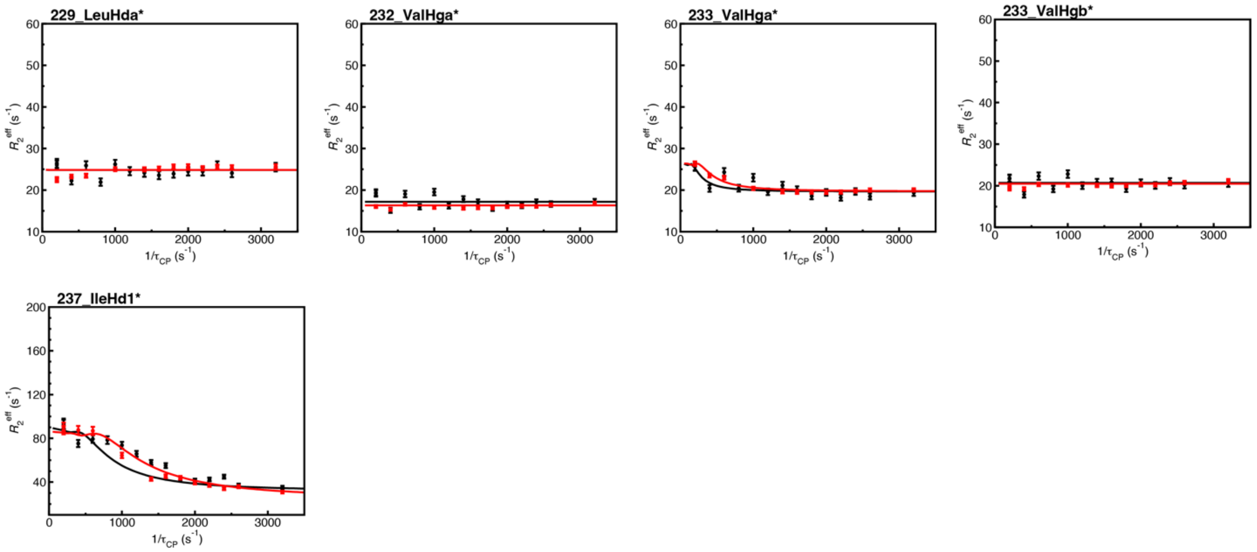

Figure S4A. R_2 relaxation dispersion profiles for the p50 DBD at 300 K, pH 6.8. Black and red, methyl ^{13}C dispersion data at 11.7 T and 18.8 T, respectively. Solid lines indicate the best fit of the individual dispersion profiles to a two-site exchange model. The designation a or b refers to the two prochiral methyl groups of Leu and Val, which have not been stereospecifically assigned.

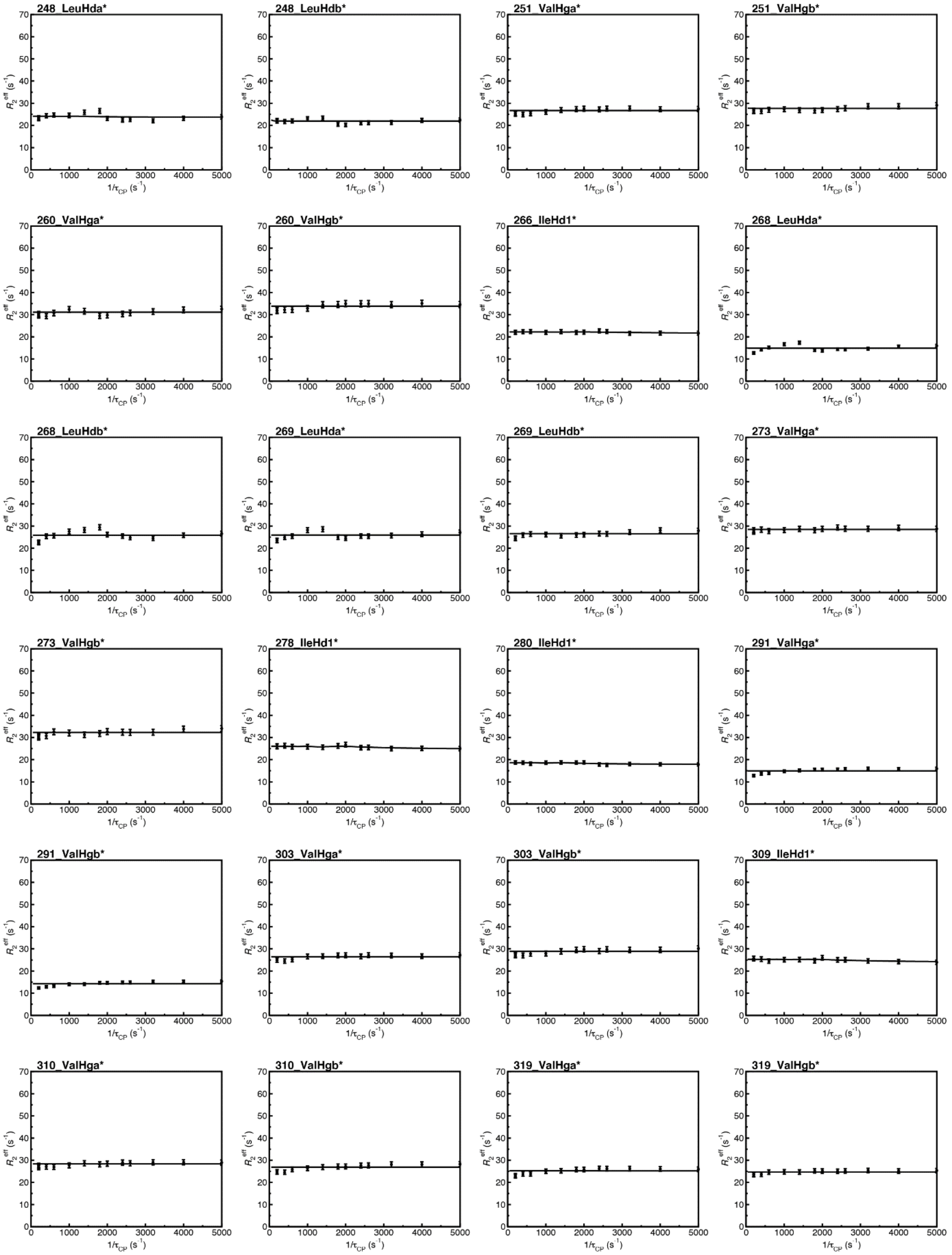
248_LeuHda*
248_LeuHdb*
251_ValHga*
251_ValHgb*
260_ValHga*
260_ValHgb*
266_IleHd1*
268_LeuHda*
268_LeuHdb*
269_LeuHda*
269_LeuHdb*
273_ValHga*
273_ValHgb*
278_IleHd1*
280_IleHd1*
291_ValHga*
291_ValHgb*
303_ValHga*
303_ValHgb*
309_IleHd1*
310_ValHga*
310_ValHgb*
319_ValHga*
319_ValHgb*
R_2^{eff} (s^{-1})
1/τ_{CP} (s^{-1})

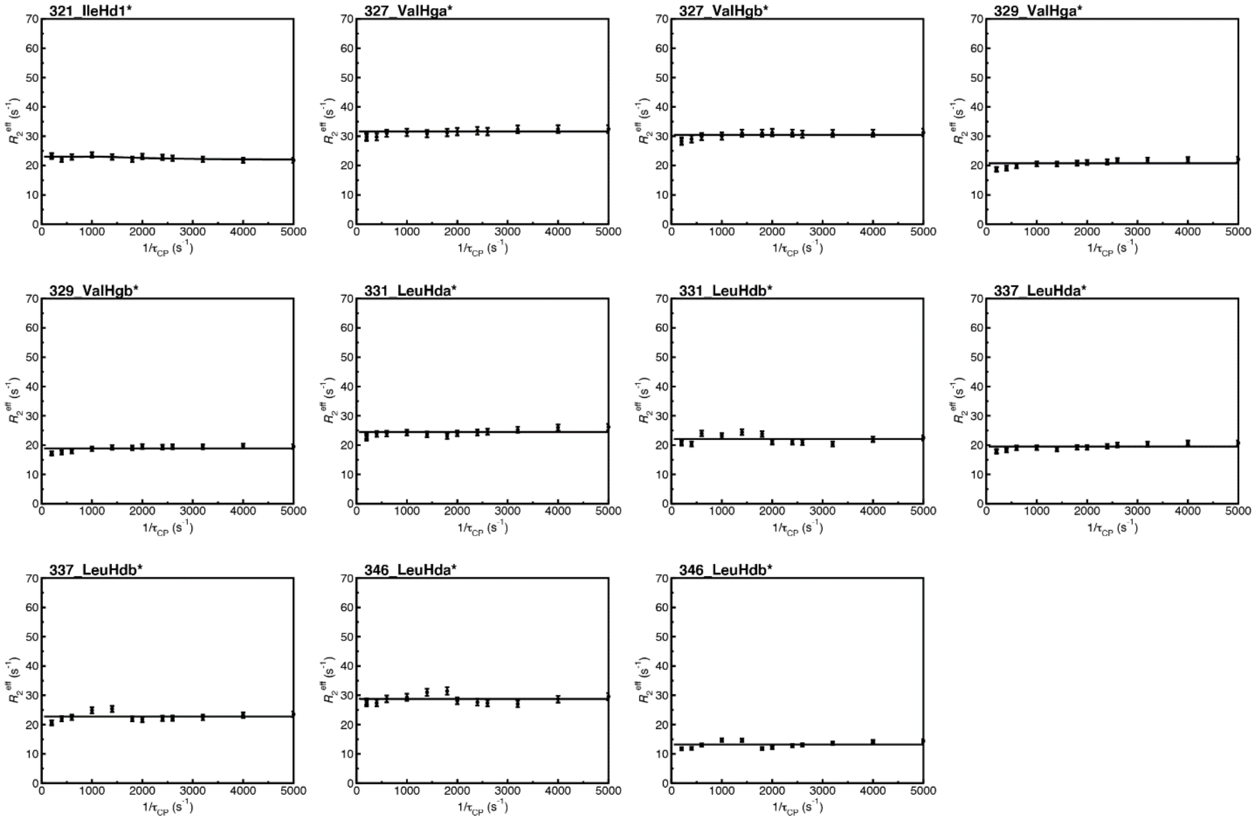

Figure S4B. R_2 relaxation dispersion profiles for the p50 dimerization domain at 300 K, pH 6.8, 18.8 T. The designation a or b refers to the two prochiral methyl groups of Leu and Val, which have not been stereospecifically assigned.

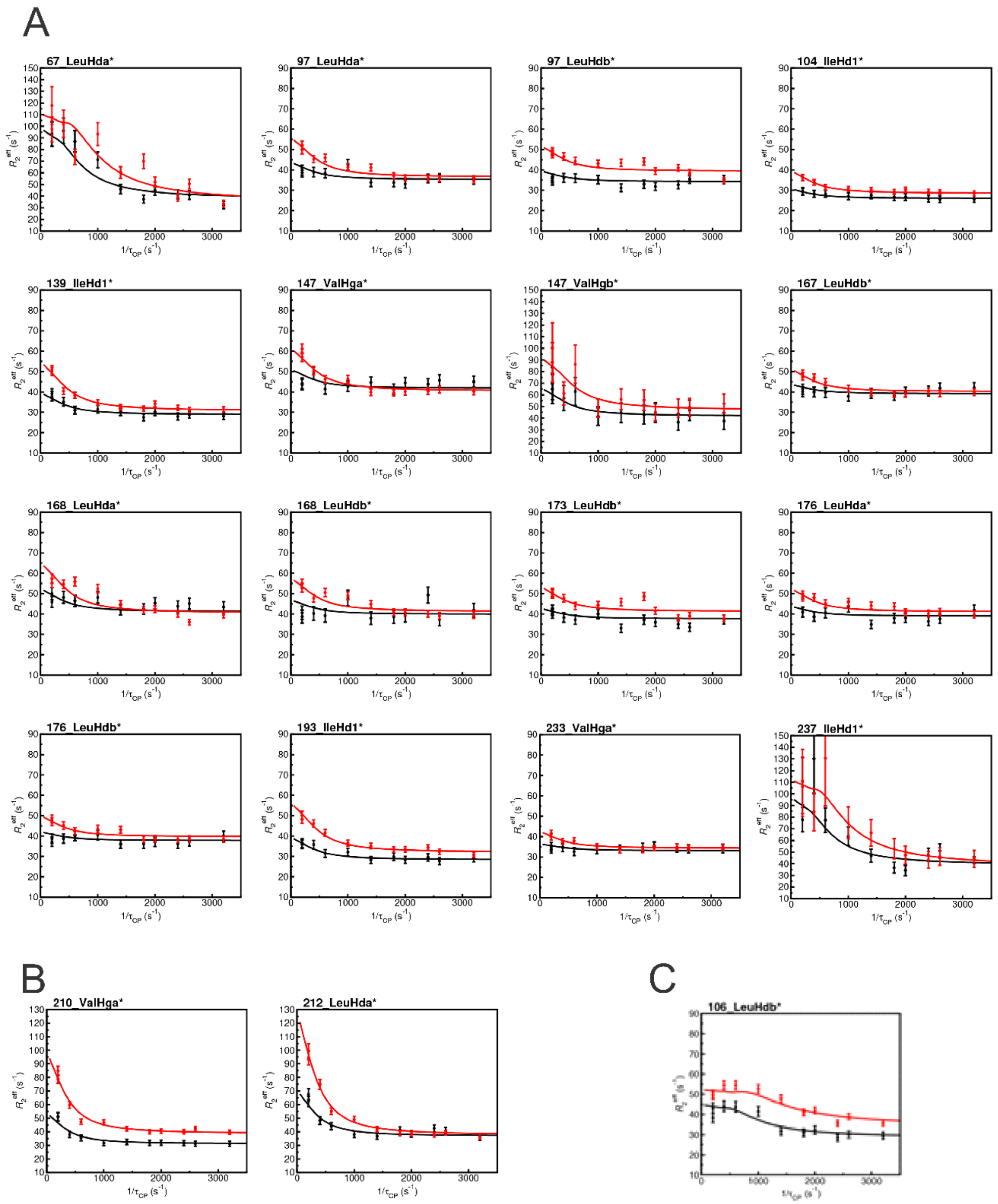

Figure S5. R_2 relaxation dispersion profiles for p50 RHR homodimer at 300 K, pH 6.8. Black and red, methyl ^{13}C dispersion data at 11.7 and 18.8 T, respectively. The designation a or b refers to the two prochiral methyl groups of Leu and Val, which have not been stereospecifically assigned. A. Cluster 1. Solid lines indicate the best fit of the data to a two-site exchange model with k_{ex} = 1600 s^{-1} and p_B = 0.051. B. Cluster 2. Solid lines indicate the best fit of the data to a two-site exchange model with k_{ex} = 660 s^{-1} and p_B = 0.14. C. Individual fit for Leu106 CδH_3(b). Solid lines represent an individual two state fit with k_{ex} = 1080 s^{-1} and p_B = 0.03.

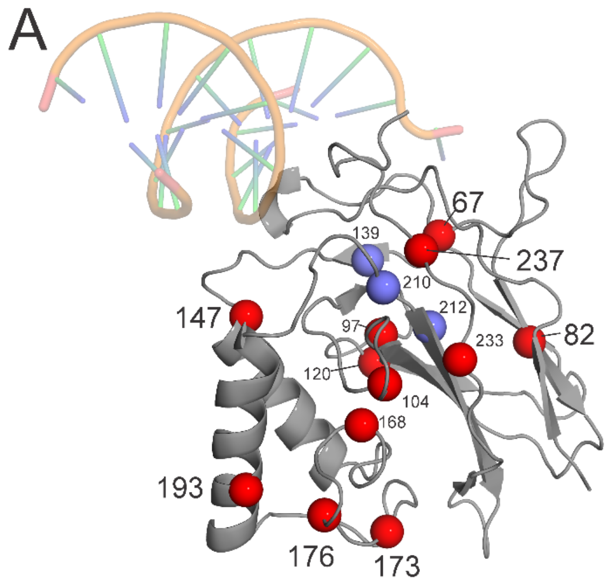

Figure S6. Comparison of the locations of dispersing residues in A. the free p50 DBD and B. the free p50 RHR (also seen in text Figure 4A). Cα positions of residues with methyls showing dispersion are indicated by spheres, red for Cluster 1 and blue for Cluster 2. The structure is a portion of the X-ray structure 1NFK. The DNA is not present, but is shown to indicate the location of the DNA binding site.

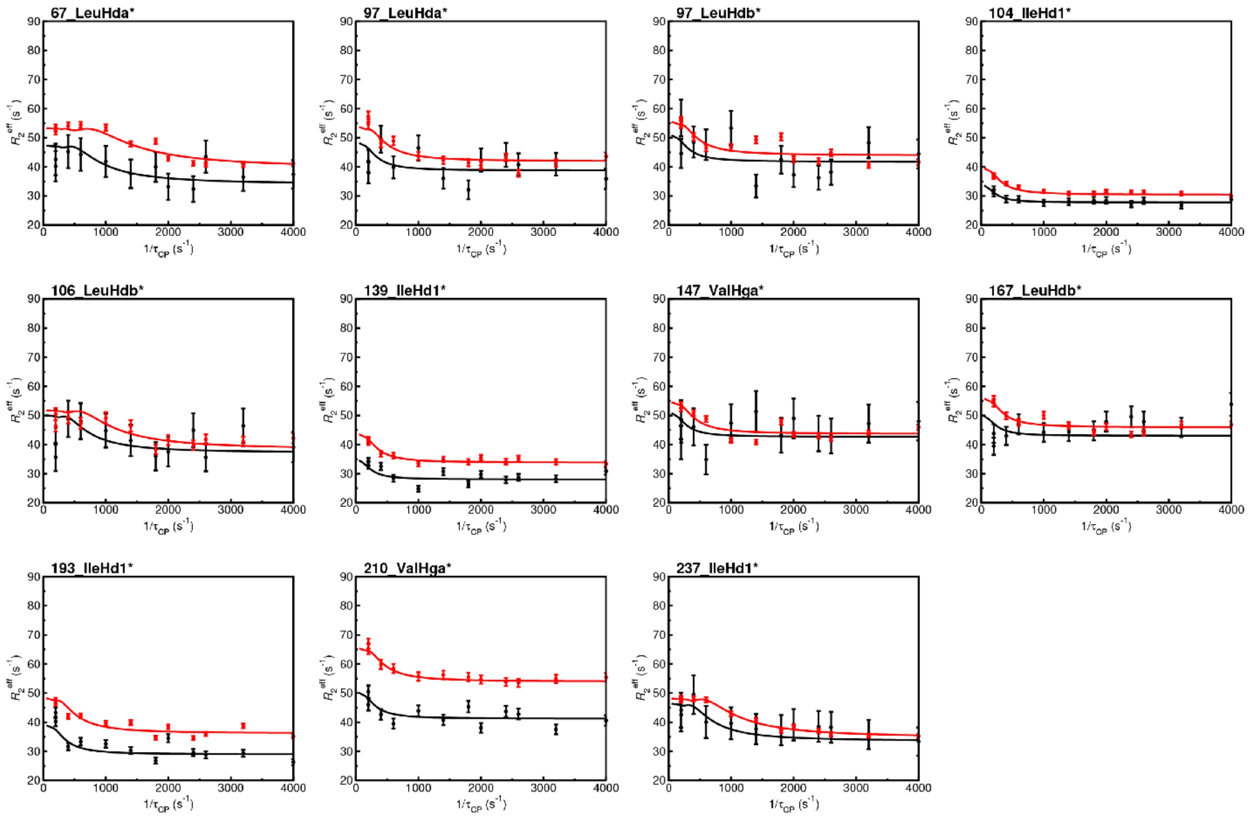

Figure S7. R_2 relaxation dispersion profiles for the p50 RHR homodimer at 290 K, pH 6.8. Black and red, methyl ^{13}C dispersion data at 11.7 and 18.8 T, respectively. Solid lines indicate the best fit of the data to a global two-site exchange model with k_{ex} = 480 s^{-1} and p_B = 0.03. The designation a or b refers to the two prochiral methyl groups of Leu and Val, which have not been stereospecifically assigned.

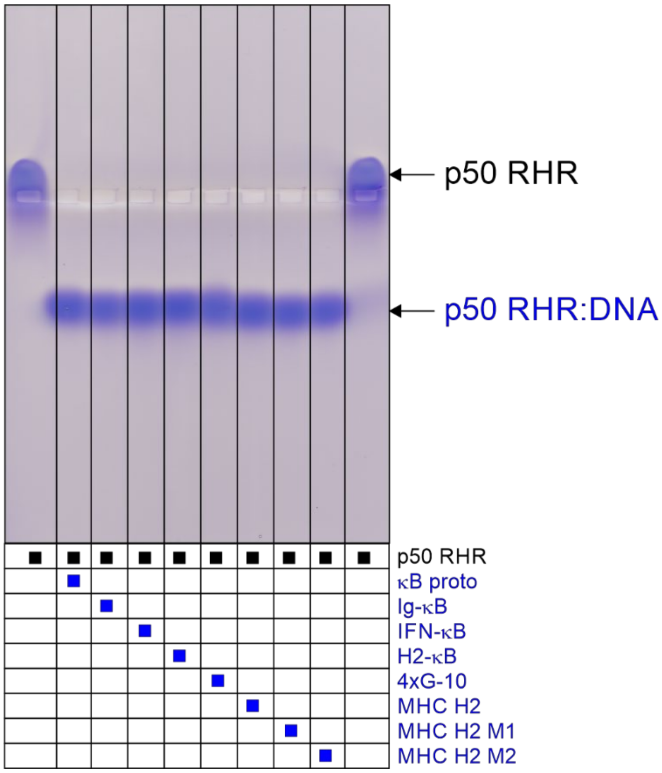

Figure S8. Native gel electrophoresis of p50 RHR and complexes with DNA oligonucleotides. Conditions were the same as previously reported (15). Briefly, samples containing 20 μM p50 RHR homodimer and DNA oligonucleotides where indicated were combined at a 1:1 ratio in 20 mM Tris, pH 8.0, 10% glycerol and incubated at room temperature for 20 min. Samples were loaded onto a 0.8% agarose gel in Tris glycine native running buffer (25 mM Tris base, 192 mM glycine, pH 8.3), electrophoresed at 50 V for 90 min, and stained with Coomassie R-250.

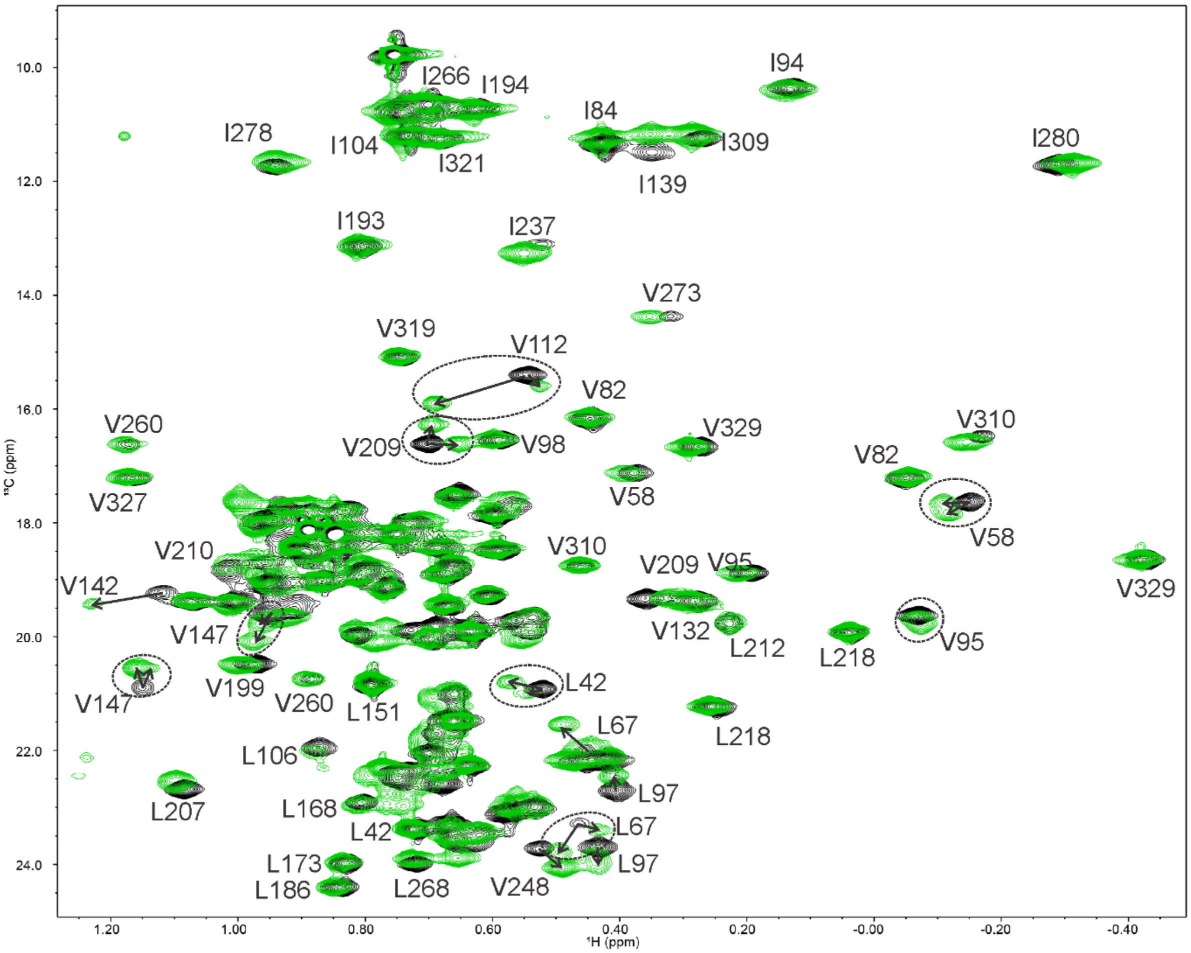

Figure S9A. Comparison of ^{13}C methyl HMQC spectra of free ILV p50 RHR (black) and in complex with the κB proto DNA duplex (green), collected at 300K, pH 6.8, 800 MHz spectrometer. A selection of resonances are labeled, and arrows show the corresponding peaks in the two spectra. Resonances that show more than one cross peak in the spectrum of the complex are circled.

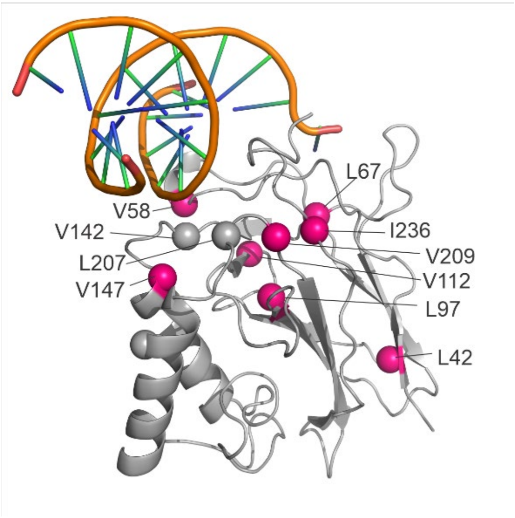

Figure S9B. Mapping of the Cα positions (spheres) of p50 DBD residues that shift upon DNA binding: only one visible resonance (gray) or one or more methyl groups show splitting of resonances in one or more of the DNA complexes (pink).

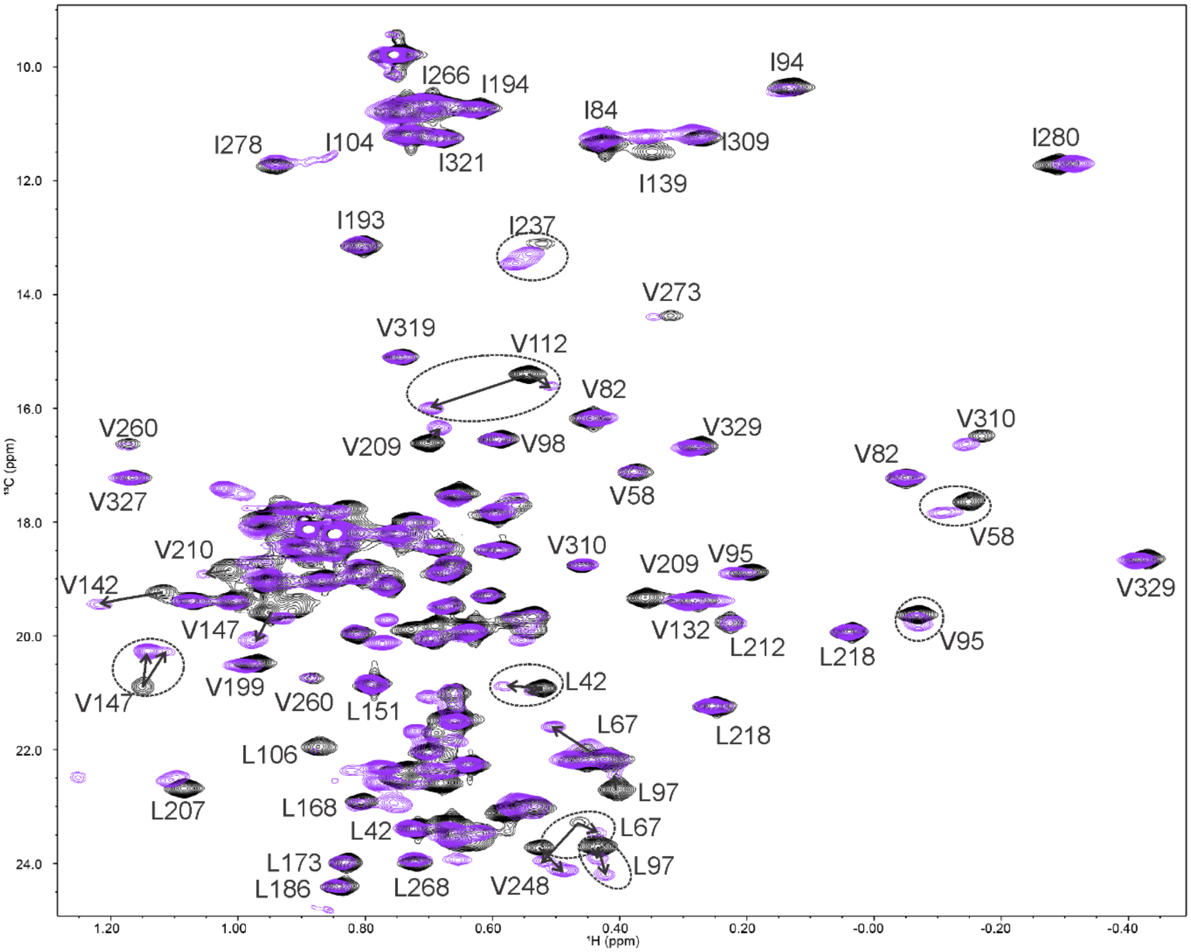

Figure S9C. Comparison of ^{13}C methyl HMQC spectra of free ILV p50 RHR (black) and in complex with the H2-κB DNA duplex (purple), collected at 300K, pH 6.8, 800 MHz spectrometer. A selection of resonances are labeled, and arrows show the corresponding peaks in the two spectra. Resonances that show more than one cross peak in the spectrum of the complex are circled.

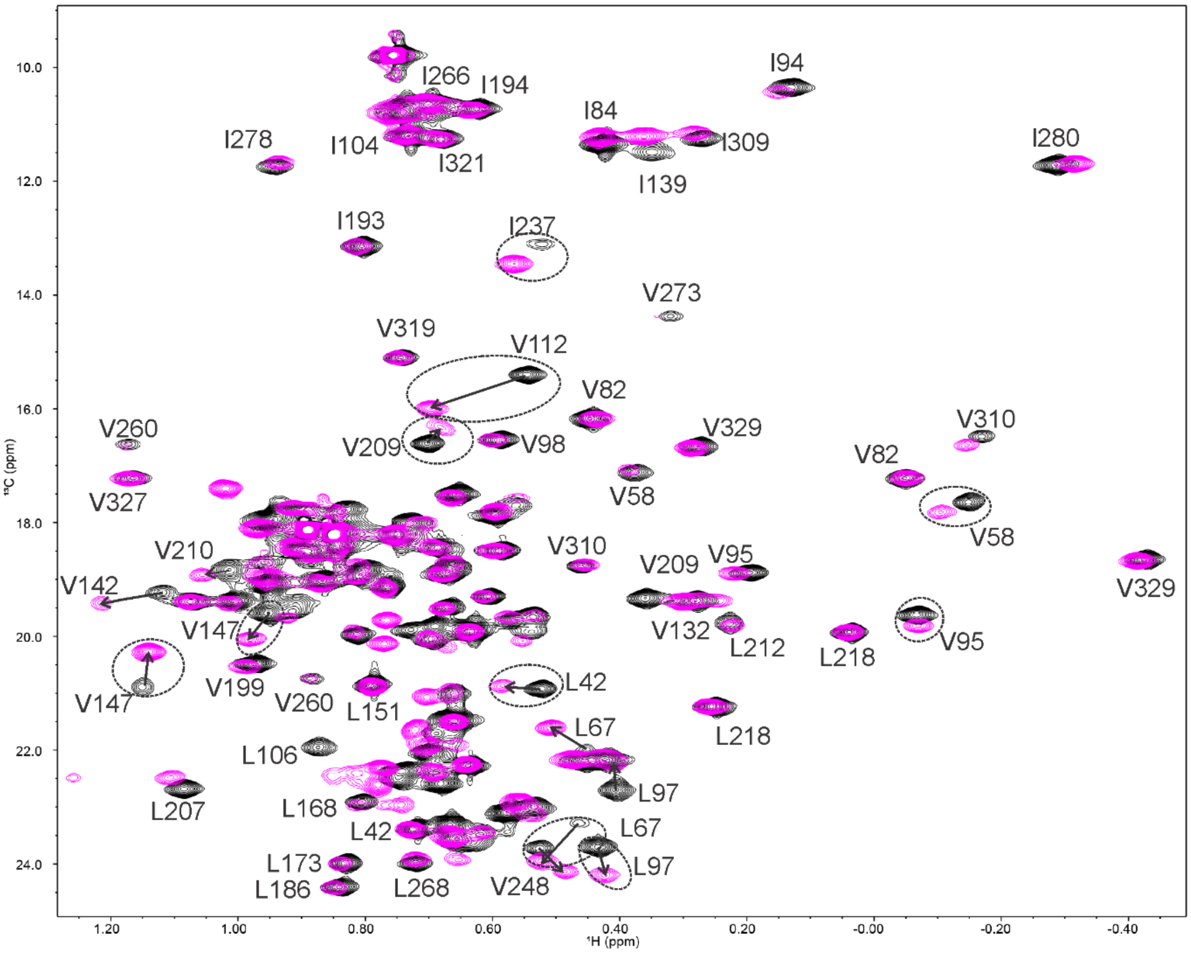

Figure S9D. Comparison of ^{13}C methyl HMQC spectra of free ILV p50 RHR (black) and in complex with the MHC H2 DNA duplex (magenta), collected at 300K, pH 6.8, 800 MHz spectrometer. A selection of resonances are labeled, and arrows show the corresponding peaks in the two spectra. Resonances that showed more than one cross peak in the spectra of the complexes in Figures 6A and/or 6B, but now show only one cross peak in the complex are circled.

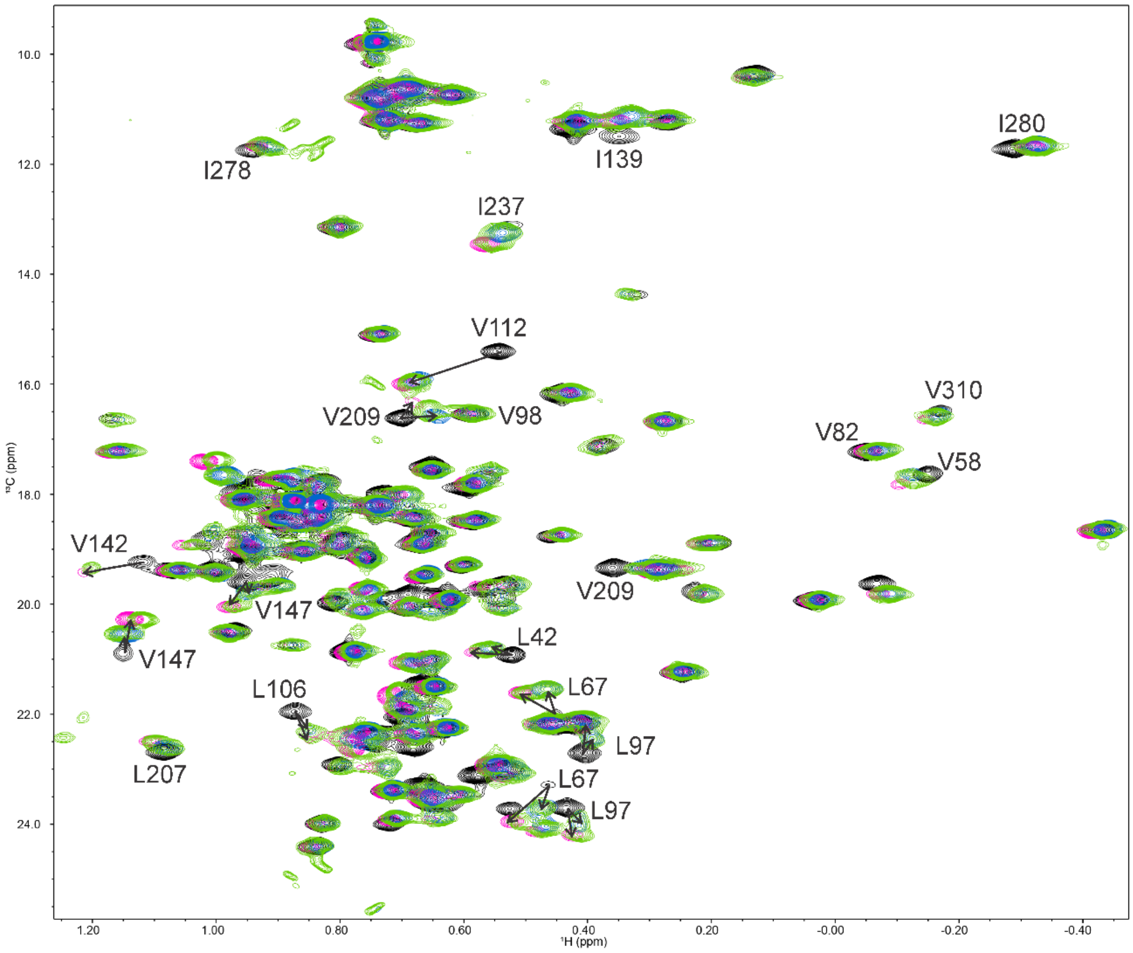

Figure S10A. Comparison of ^{13}C methyl HMQC spectra of free ILV p50 RHR (black) and in complex with the DNA duplexes MHC H2 (magenta), MHC H2M1 (blue) and MHC H2M2 (green). Spectra were collected at 300K, pH 6.8, 800 MHz spectrometer. A selection of resonances are labeled, and arrows show the corresponding peaks in the four spectra.

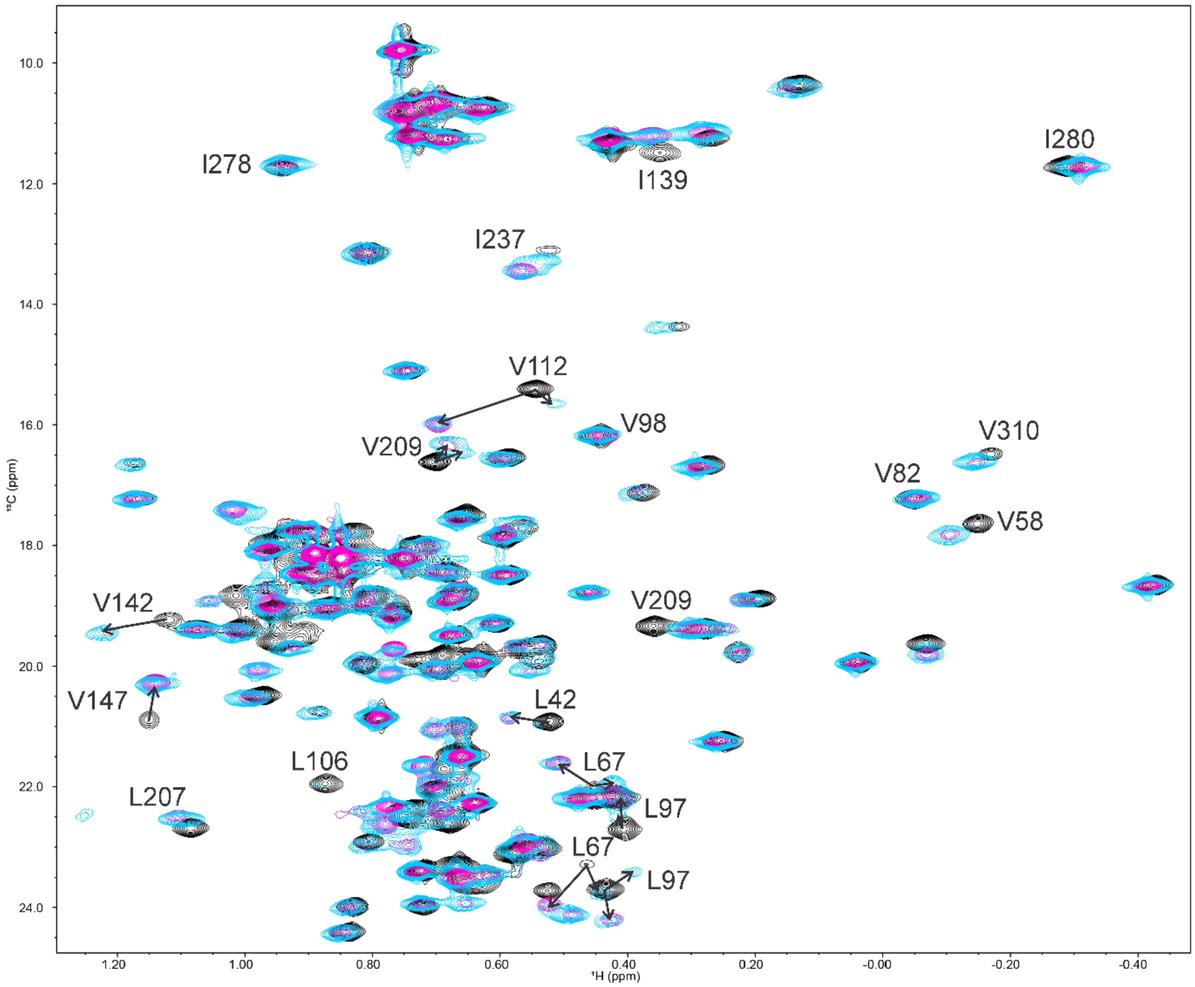

Figure S10B. Comparison of ^{13}C methyl HMQC spectra of ILV p50 RHR free (black) and in complex with the 11bp DNA duplex MHC H2 (magenta) and the 10bp duplex 4xG-10 (cyan). Spectra were collected at 300K, pH 6.8, 800 MHz spectrometer.

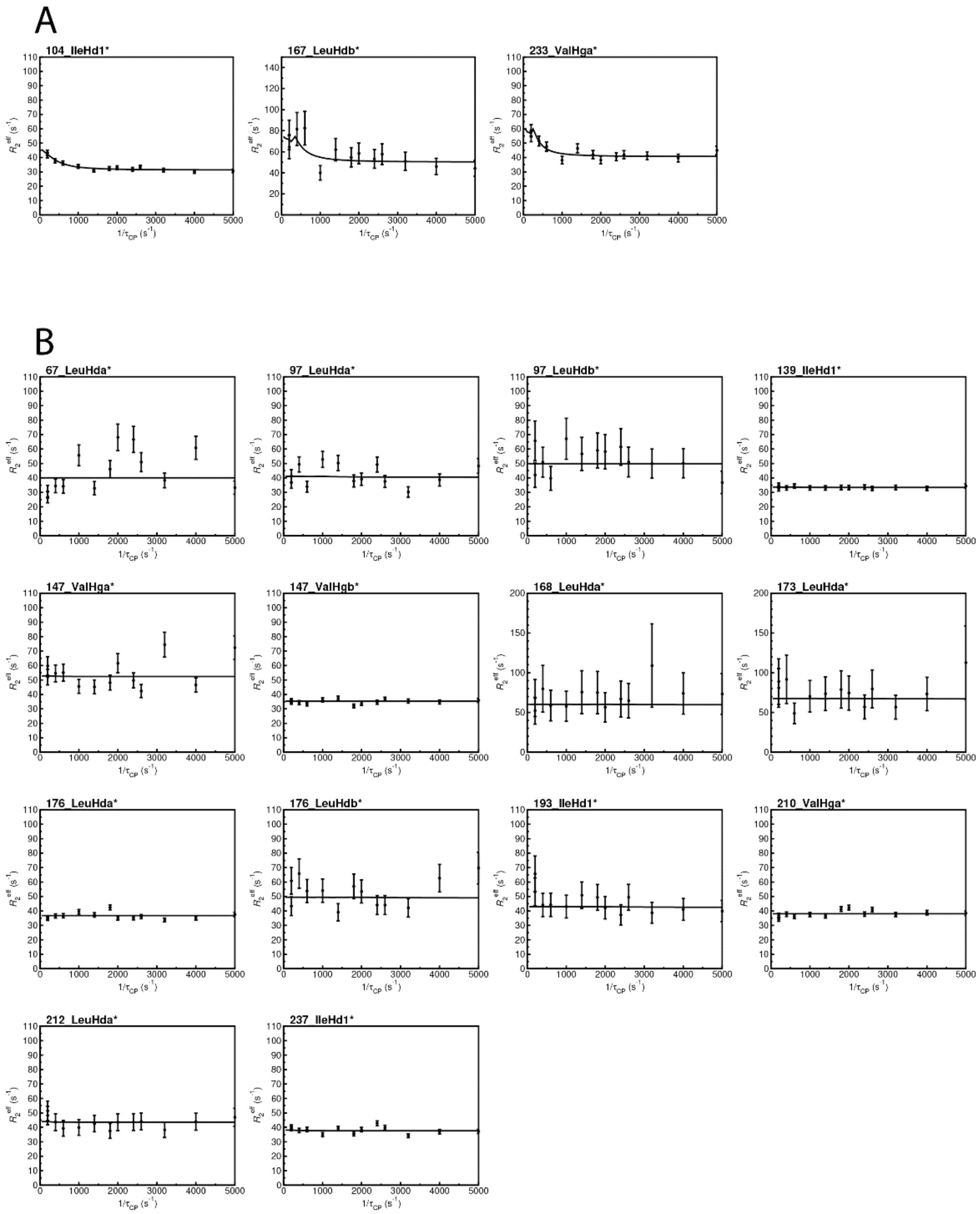

Figure S11. R_2 relaxation dispersion profiles for 250 μM p50 RHR homodimer at 300 K, pH 6.8 in the presence of an equimolar concentration of MHC H2 DNA. Black curves show methyl ^{13}C dispersion data at 18.8 T. The designation a or b refers to the two prochiral methyl groups of Leu and Val, which have not been stereospecifically assigned. A. Residues showing dispersion in the DNA-bound state. B. Residues showing no dispersion in the DNA-bound state.
